# Steroidal A/B-ring fusion as a strategy for isoform-selective inhibition of human carbonic anhydrases

**DOI:** 10.1039/d5ra06507k

**Published:** 2026-02-18

**Authors:** Jiří Brynda, Anita Kiss, Klára Pospíšilová, Vojtěch Kapras, Irena Sieglová, Barbora Slavíková, Pavlína Řezáčová, Eva Kudová

**Affiliations:** a Institute of Organic Chemistry and Biochemistry, Czech Academy of Sciences Prague Czech Republic kudova@uochb.cas.cz

## Abstract

Carbonic anhydrases (CAs) are zinc-containing metalloenzymes that catalyse the reversible hydration of carbon dioxide and thus play a crucial role in pH regulation. Among the isoforms of carbonic anhydrases, CA IX is a cancer-associated enzyme overexpressed in hypoxic tumours, which makes it an attractive target for anti-cancer drug development. Steroidal compounds, with their rigid frameworks and diverse functionalization potential, have emerged as promising scaffolds for designing selective CA inhibitors. Their ability to engage in specific enzyme interactions makes them valuable for developing selective inhibitors targeting medically relevant CA isoforms. This study explores the inhibitory activities and binding modes of four steroid skeletons—5β-steroid, estra-1,3,5(10)-triene, Δ5-steroid and 5α-steroid—in interaction with selected CA isoforms. Structural and inhibition studies have revealed that steroidal sulfamate derivatives effectively coordinate with the active site zinc ion, adopting distinct binding modes based on isoform-specific variations. The hydrophobic patch at the active site entrance, influenced by a key difference in the residue present at position 131 (Phe131 in CA II *vs.* Val131 in CA IX), plays a crucial role in modulating binding interactions. Estra-1,3,5(10)-triene derivatives exhibit nanomolar inhibition of both CA II and CA IX, demonstrating adaptability through alternative binding conformations. By contrast, Δ5-steroid compounds show enhanced selectivity towards CA IX and appear to be less easily accommodated by the more constrained active site of CA II. These findings highlight the potential of steroidal compounds as inhibitors of specific CA isoforms. In particular, estra-1,3,5(10)-triene and Δ5-steroid compounds without a C-17 substitution emerge as strong candidates for further development, targeting the cancer-associated CA IX and other medically relevant isoforms.

## Abstract 2

Steroidal sulfamates inhibit cancer-related CA IX by binding its zinc active site. Estra-1,3,5(10)-triene and Δ5-steroids show strong, selective activity, highlighting their potential as targeted anticancer agents.

## Graphical abstract

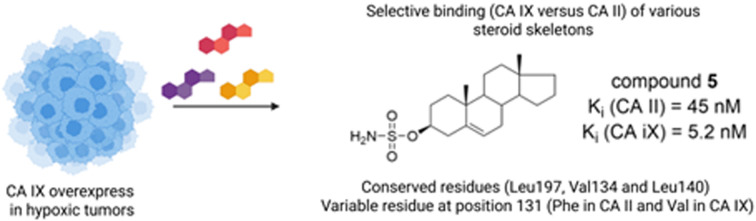

## Introduction

Carbonic anhydrases (CAs) are zinc-containing metalloenzymes that catalyse the reversible hydration of carbon dioxide into bicarbonate and protons. In humans, fifteen distinct isoforms have been identified, each differing in its expression patterns and tissue distribution.^1^ These isoforms, however, exhibit a high degree of amino acid sequence homology.^2^ Given their role in a wide range of physiological and pathological processes, CA inhibitors have been extensively explored as potential therapeutics for conditions such as cancer,^3–5^ glaucoma,^6–8^ epilepsy,^9^ diuretics,^10,11^ and obesity.^12–14^ In this study, we focus on the inhibition of two CA isoforms: the ubiquitously expressed CA II isoform and the tumour-associated CA IX isoform. To study this inhibition, we employed an array of steroid-based molecules as molecular probes that allowed us to describe recognition features essential for developing selective steroidal CA inhibitors. Several recent reviews^15–19^ have provided comprehensive updates on the development of CA IX inhibitors, highlighting notable progress in the design of selective inhibitors, their potential applications in cancer therapy, and the challenges that remain to be addressed (Table 1). The field is evolving rapidly, driven by new structural insights, a deeper understanding of CA IX biology, and the emergence of promising clinical candidates that are advancing toward effective and selective CA IX-targeted therapies. Among the clinically investigated inhibitors, SLC-011, a sulfonamide-based small molecule with high selectivity for CA IX and CA XII, represents the most advanced compound.^20–23^ It has completed Phase I and is currently undergoing Phase Ib/II clinical trials for the treatment of advanced hypoxic solid tumors.^24^ Classical sulfonamides such as acetazolamide,^25,26^ methazolamide, ethoxzolamide, dorzolamide, and brinzolamide also inhibit CA IX *in vitro*;^27^ however, these compounds lack isoform selectivity and are not approved specifically for CA IX-related cancer therapy.^28^ In addition to clinically evaluated inhibitors, numerous novel chemical scaffolds – including triazolopyrimidines,^29^ carborane-based sulfonamides,^30,31^ benzenesulfonamides,^32^ uracil/adenine analogues, and coumarin derivatives^33,34^ – have demonstrated potent and selective CA IX inhibition. Only the most recent examples of these compound classes are highlighted here (Table 1), as they illustrate current strategies and trends in the design of potent and isoform-selective CA IX inhibitors. Despite their promising activity and mechanistic diversity, none of these compounds have yet progressed to clinical development for cancer therapy.

**Table 1:** Overview of inhibitors of CA IX

Drug	Structural type	Name	*K*_i_ value CA II (nM)	*K*_i_ value CA IX (nM)	Selectivity index CA II *K*_i_/CA IX *K*_i_
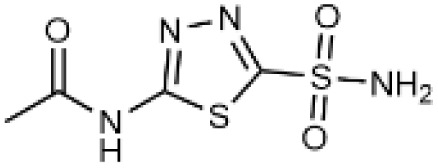	Sulfonamides	Acetazolamide	12	25	0.48
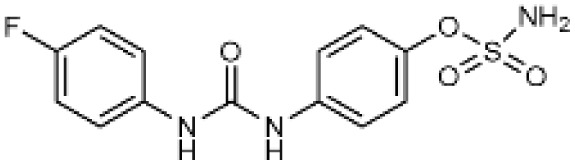	Ureido-substituted benzenesulfonamides	SLC-0111	960	45	21
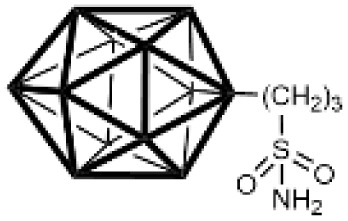	Sulfonamido carboranes		622	0.506	1229
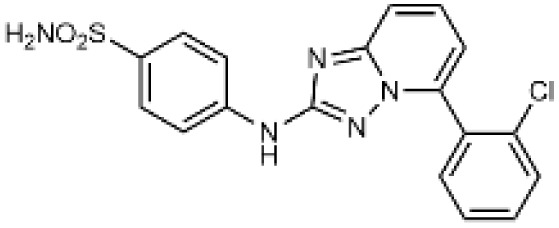	Triazolopyrimidines		155	15	10
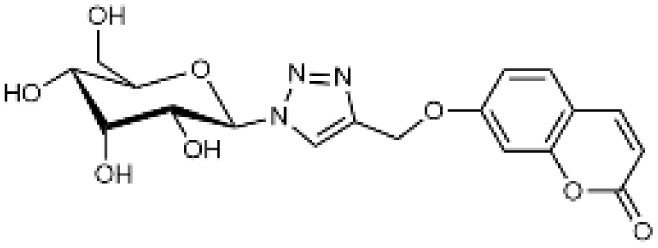	Substituted coumarins		21 500	11	1954
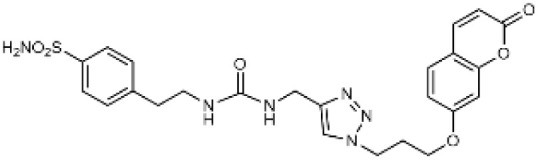	Substituted coumarins		61	6	10
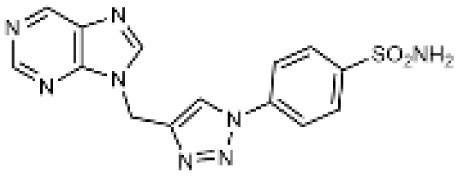	Adenine derivatives		5.6	1.9	2.9
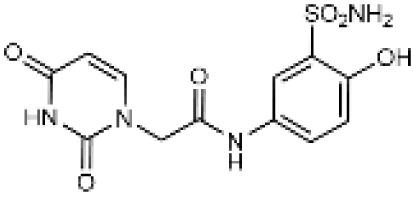	Uracil derivatives		4357	439	10
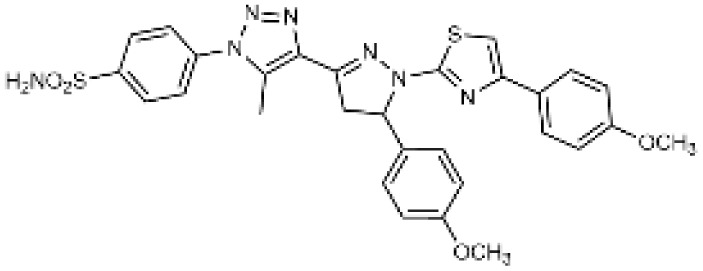	Benzensulfonamide		95	25	3.8
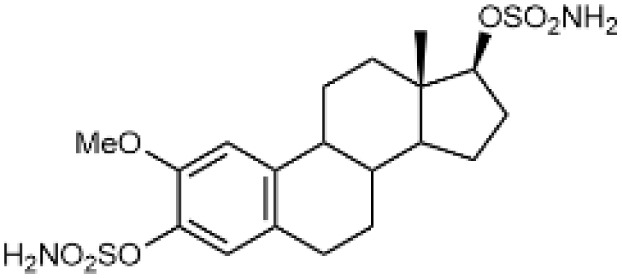	Estrone sulfamates	STX140	270	70	3.9
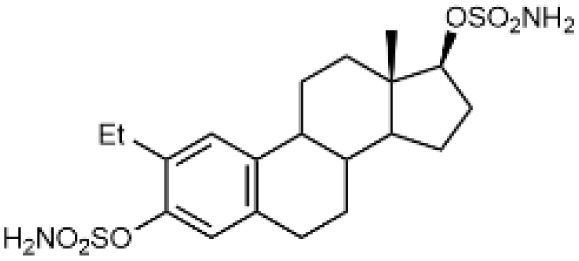	Estrone sulfamates	STX243	2420	250	9.6
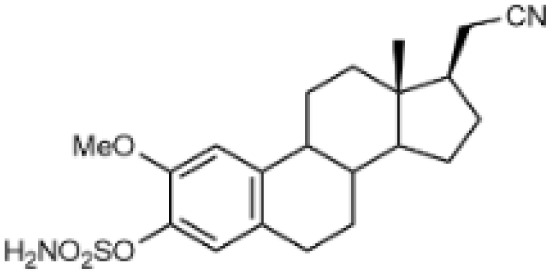	Estrone sulfamates	STX641	1470	750	1.9
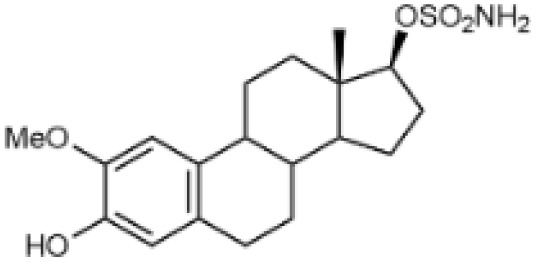	Estrone sulfamates	STX738	10 330	1180	8.7

Steroidal sulfamate derivatives, such as 3,17β-bis-sulfamoyloxy-2-methoxyestra-1,3,5(10)-triene (STX140) and its analogues STX243, STX641, and STX738,^35^ have been identified as inhibitors of CA IX. These compounds are based on modifications of endogenous steroid hormone 2-methoxyestradiol. Several strategies have been reported for designing selective CA IX inhibitors,^35–37^ including ones based on a dual-target approach aimed at inhibiting the activity of aromatase and/or steroid sulfatase.^38^

Although many novel inhibitors show strong affinity towards specific CA isoforms, their potential clinical use is limited by poor selectivity, leading to a broad range of side effects. Addressing this issue requires an understanding of the differences between the active sites of various isoforms, which would enable the development of ligands that would selectively bind to specific target sites. This can be achieved by taking an integrated approach combining structure–activity relationship (SAR) studies with high-resolution structural data to elucidate isoform-specific binding interactions.

**Fig. 1:**
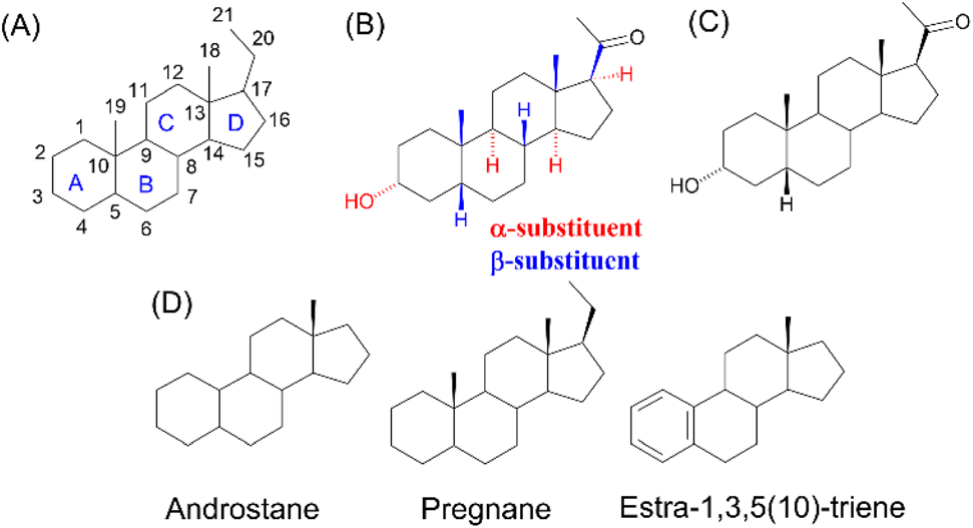
(A) Numbering and lettering of the rings of steroid compounds. (B) Absolute stereochemistry defined for all chiral stereocentres: α-substituents (hashed bond lines) and β-substituents (bold bond lines). (C) Simplified stereochemistry of steroid molecules; (D) trivial names of steroid skeletons relevant to this article.

Steroids are endogenous molecules characterized by a cyclopenta[*a*]phenanthrene skeleton. Their four-ring system is labelled and numbered as shown in Fig. 1A. The three-dimensional structure of this scaffold is defined by stereochemistry at eight stereocentres: C-3, C-5, C-8, C-9, C-10, C-13, C-14 and C-17. By convention, the configuration of substituents is represented using hashed (α) or bold (β) bond lines (Fig. 1B), indicating their orientation relative to the plane of the steroid ring. Natural steroids predominantly adopt the 8β-H, 9α-H, 10β-CH_3_, 13β-CH_3_, 14α-H and 17α-H stereochemistry (Fig. 1B). Simplified representations (Fig. 1C) help illustrate these stereochemical elements, particularly at positions C-3 and C-5, which are crucial for defining the molecular conformation.

**Fig. 2:**
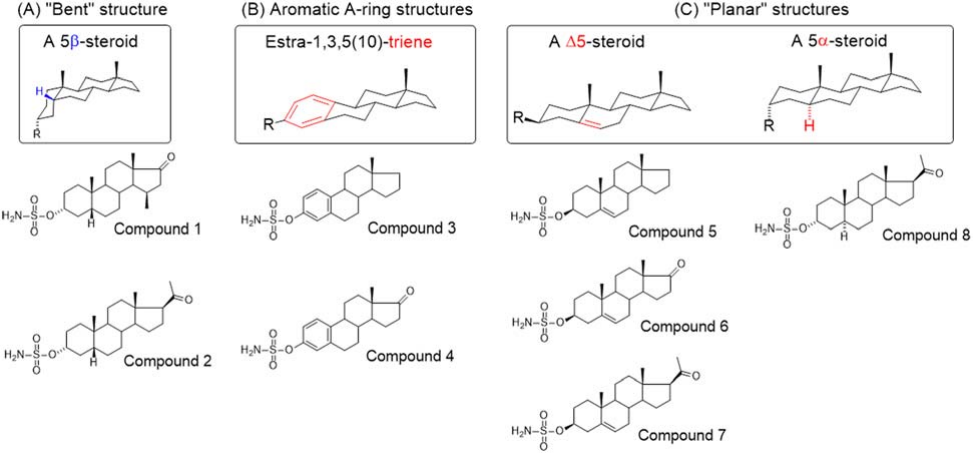
(A) Perspective view of a curved molecule of a 5β-steroid with examples of two such compounds. (B) Perspective view of structures with an aromatic A-ring, namely two estra-1,3,5(10)-trienes. (C) Perspective view of planar three Δ5-steroids and one 5α-steroid.

Steroidal *O*-sulfamates commonly feature an aromatic A-ring with an *O*-sulfamate moiety, as exemplified by compound 4 (Fig. 2B).^38^ Recognizing the importance of pharmacophores in drug design, we explored the role of the aromatic A-ring and substituents at position C-17 in modulating activity against CA II, CA VII and CA IX. Specifically, our study examines the steroidal C-5 configuration (*i.e.* the fusion of the A- and B-rings) as a tool to probe the steric and spatial requirements of the CA active site.

The fusion of the A- and B-rings defines the shape of the molecule (Fig. 2). Steroids with *cis*-fused A/B-rings, such as compounds 1 and 2, adopt a ‘bent’ conformation, where the C-5 proton and C-10 angular methyl are oriented adjacent to each other (compounds 1 and 2). By contrast, compound 8 (an 5α-steroid) features a trans conformation of the A- and B-rings, where the C-5 proton and C-10 angular methyl are oriented opposite to each other. For this reason, this steroid skeleton is referred to as being ‘planar’. However, considering the shape of this skeleton, the definition of a ‘planar’ steroid can also be applied to any Δ5 steroid (compounds 5, 6 and 7), which have a double bond between C-5 and C-6.

Steroids with an aromatic A-ring (3 and 4) cannot adopt a fully planar shape, because the estra-1,3,5(10)-triene skeleton does not allow the trans conformation of the A- and B-rings and are therefore considered ‘semi-planar’ in this study.

It is important to note that the axial or equatorial orientation of substituents in steroid structures is fixed due to the rigid structure of the *cis*- or *trans*-fused rings. In particular, the stereochemistry of the C-3 and C-5 substituents determines the global shape of the steroid skeleton, including its substituents and their position. Note that the 3α-substituent of the planar 5α-H skeleton is axial whereas the 3α-substituent of the ‘bent’ 5β-H skeleton is equatorial.

**Fig. 3:**
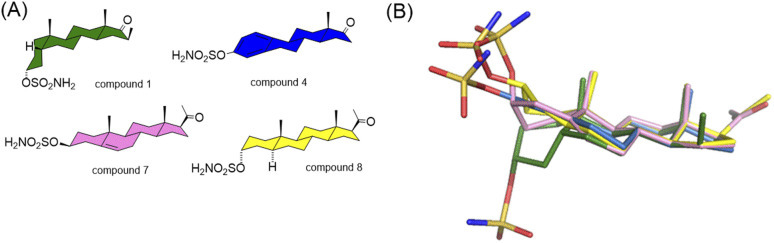
Structural formulas (A) and 3D structures (B) of steroid compounds 1 (a curved 5β steroid, green), 4 (an aromatic steroid, blue), 7 (a Δ5 steroid, pink) and 8 (a planar 5α steroid, yellow).

Due to the conformational flexibility inherent in steroidal molecules, even a single modification—such as an altered stereochemistry at C-5—can significantly affect the shape of the molecule (Fig. 2). This variability makes steroids an excellent class of compounds for probing the steric landscape of enzyme active sites like that of carbonic anhydrases. Fig. 3 presents examples of a curved 5β-steroid (compound 1), an aromatic steroid (compound 4), a Δ5-steroid (compound 7) and a planar 5α-steroid (compound 8), demonstrating the diverse spatial conformations made possible by the steroidal scaffold.

## Results and discussion

### Chemistry

**Scheme 1:**
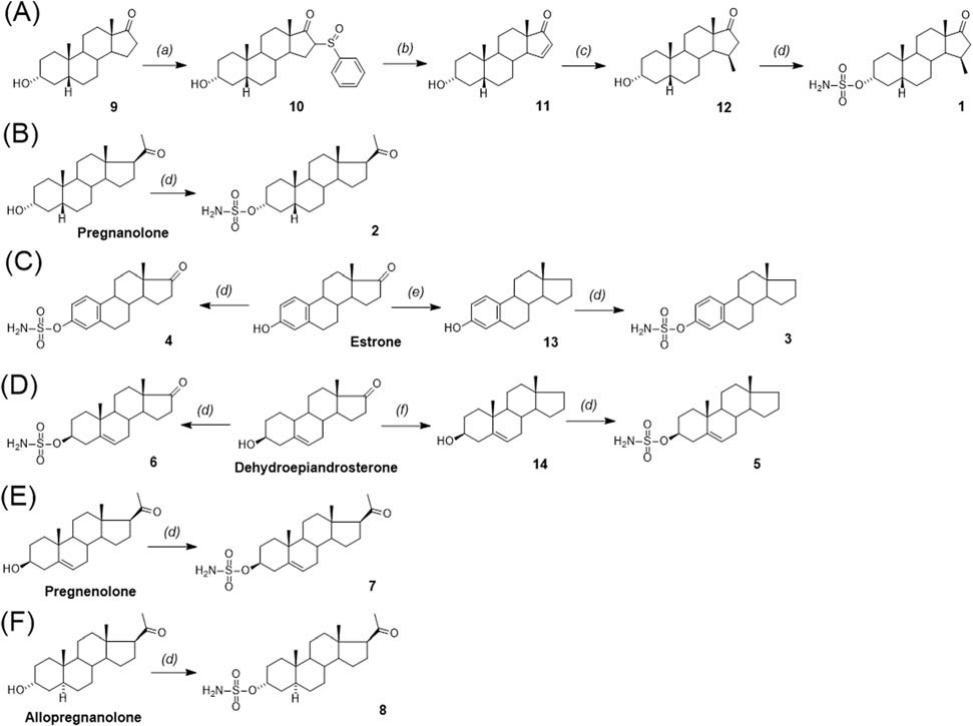
Synthesis of compounds 1–8. Reagents and conditions: (a) methyl phenylsulfinate, *t*BuOK, THF, 25–30 °C; (b) Na_2_CO_3_, xylene, 140 °C; (c) CuI, MeMgBr, THF, toluene; (d) sulfamoyl chloride, *N*,*N*-dimethyl acetamide, rt; (e) hydrazine monohydrate, potassium hydroxide and diethylene glycol; (f) Zn, TMSCl, methanol, dichloromethane.

The synthesis of sulfamates 1–8 is summarized in Scheme 1. The compounds were prepared by the sulfamoylation of the corresponding steroid alcohols by treatment with sulfamoyl chloride in the presence of *N*,*N*-dimethylacetamide.^39^ Sulfamate 1 was prepared by multi-step synthesis from 3α-hydroxy-5β-androstan-17-one (9) through sulfinylation.^40,41^ First, the α-sulphoxide substituent (10) was introduced by a reaction of 9 with methylphenylsulfinate in the presence of potassium *tert*-butoxide. Thermal elimination under reflux with sodium carbonate then provided the unsaturated product 11. Finally, alkylation to the α,β-unsaturated compound 11, promoted by copper iodide, afforded the 15β-methylated compound 12. Sulfamate 1 was prepared from compound 9 in a total yield of 7% after four reaction steps. Sulfamates 2, 4 and 8 were prepared from the parent 3-hydroxy compounds pregnanolone, pregnenolone, and estrone by following procedures described in the literature.^42,43^ Sulfamate 3 was prepared from commercially available estrone by the Huang–Minlon modification of Wolff–Kishner decarbonylation^44^ of steroid ketones with hydrazine hydrate and diethylene glycol to give compound 13. This was followed by sulfamoylation to compound 3 with a yield of 56%. Sulfamate 5 was analogously prepared from 3β-hydroxy-androst-5-ene (14), which was obtained by the Zn/TMSCl-mediated Clemmensen reduction^45^ of dehydroepiandrosterone. Sulfamates 6 and 7 were prepared from pregnenolone and allopregnanolone by sulfamoylation with sulfamoyl chloride in 48% and 73% yield, respectively.

### Inhibitory activity

We evaluated the target steroidal compounds for their ability to inhibit recombinant enzymes *in vitro* by conducting a stopped-flow carbon dioxide hydration assay. Our study focused on comparing their inhibitory effects on two CA isoforms: the widely expressed isoform CA II and the cancer-associated isoform CA IX. Despite the high level of conservation of their active sites, limited amino acid variability exists that can influence inhibitor affinity and confer selectivity toward one isoform. All the tested compounds effectively inhibited carbonic anhydrase activity, exhibiting *K*_i_ values in the nanomolar range (Table 2). Notably, compounds 3 and 4, both belonging to the estra-1,3,5(10)-triene class, demonstrated the strongest inhibition, with *K*_i_ values in low nanomolar range for both isoforms. The highest selectivity for the cancer-specific isoform CA IX was observed with Δ5 steroid derivatives; compounds 6 and 7 had selectivity indices close to 5, while compound 5 was over eight times more selective towards CA IX than towards CA II.

**Table 2:** *In vitro* inhibition of selected carbonic anhydrase isoenzymes

Compound	CA II *K*_i_ [nM]	CA IX *K*_i_ [nM]	Selectivity index^aa^	CA VII *K*_i_ [nM]
1	8.2 ± 0.8	32.1 ± 2.3	0.3	277.0 ± 27.4
2	16.7 ± 1.7	10.5 ± 1.7	1.6	210.4 ± 22.1
3	2.6 ± 0.3	2.5 ± 0.4	1.0	12.8 ± 1.1
4	4.5 ± 0.3	4.4 ± 0.6	1.0	17.9 ± 4.8
5	44.9 ± 2.8	5.2 ± 0.6	8.5	132.7 ± 24.6
6	73.3 ± 6.5	15.0 ± 1.8	4.9	209.2 ± 23.3
7	158 ± 15	26.4 ± 4.7	5.9	343.6 ± 25.8
8	48.4 ± 8.5	17.8 ± 1.7	2.7	309 ± 40.1
**AZA^b^**	**12.6 ± 2.0**	**15.0 ± 2.5**	**0.8**	**25.4 ± 2.3**
**U104^c^**	**20.4 ± 1.4**	**18.1 ± 1.1**	**4.9**	**357 ± 41**

a Selectivity index is the ratio between *K*_i_ (CA II) and *K*_i_ (CA IX). Purity of compound 1 was 92%. Two known CA inhibitors were used as controls.

b Acetazolamide (AZA).

c 4-[[[(4-Fluorophenyl)amino]carbonyl]amino]benzenesulfonamide (U104).

Based on the structural analysis described below, we proposed that estra-1,35(10)-triene can adapt to different shapes of the active site, with the amino acid residue at position 131 playing a crucial role in accommodating steroidal skeletons. We therefore extended our inhibition studies to isoform CA VII, the brain-specific isoform containing a bulky phenylalanine residue at position 131. All compounds exhibited significantly lower inhibitory activity against CA II than against the other two isoforms; however, compounds 3 and 4 still maintained inhibition in the double-digit nanomolar range.

### Crystal structures

We carried out structural studies to compare how the target inhibitor compounds interact with the active sites of the CA II and CA IX enzymes. To this end, we obtained the co-crystal structures for all enzyme–inhibitor complexes with CA II and CA XI mimic, a CA II enzyme containing seven amino acid residue substitutions in its active site (A65S, N67Q, E69T, I91L, F130V, K169E and L203A). This variant is often used in structural studies because it retains the good crystallization properties of CA II while having an active site similar to CA IX.^46^

The crystal structures were determined with a very high resolution of 1.3 to 1.5 Å and provided a solid basis for understanding the observed inhibitory properties and selectivity. All crystals belonged to the monoclinic *P*2_1_ crystal space group and contained one enzyme molecule in the asymmetric unit. The inhibitor compounds were clearly modelled in a well-defined active site electron density map (Fig. S4 and S5, SI). The only exception was compound 6 in the active site of CA II, where parts of the electron density map were very weak because of the high degree of flexibility of the steroid backbone, resulting in dynamic disorder (Fig. S4, SI).

**Fig. 4:**
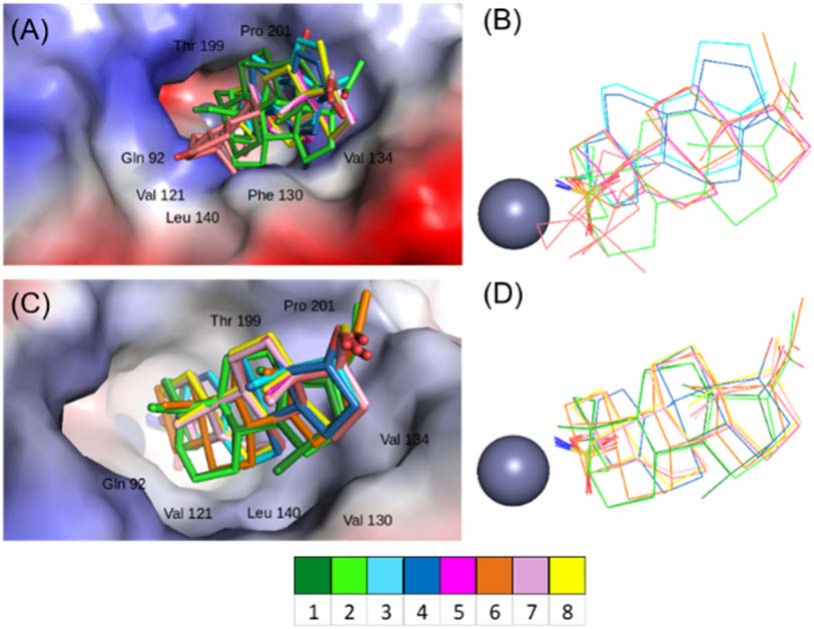
Superposition of active-site-bound steroid compounds in complex with CA II (A and B) and CA IX mimic (C and D). The superimposed compounds are shown as sticks, and the protein is represented by its solvent-accessible surface (coloured according to the electrostatic potential). Important interacting residues are indicated.

Comparative analysis of all crystal structures revealed that the sulfonamide moiety is deeply buried in the active site, where it forms polar interactions with the zinc ion and residues located at the bottom of the active site cavity (His94, His96 and His119). This binding mode is similar to active site interactions reported for other sulfonamide inhibitors.^47–49^ In both enzymes, the steroid skeletons of the inhibitor compounds studied here are oriented towards the hydrophobic region of the active site funnel (Fig. 4) and form numerous strong hydrophobic interactions with the residues Gln92, Val121, Phe130 (or Val 130 in the CA IX active site), Val134, Thr199 and Pro201.

The binding mode of inhibitors in the active site of CA IX is quite conserved. The compounds tested assume a similar orientation, with atom C-15 of the D-ring pointing towards C_alpha_ of Val130. The main difference resides in the torsion angle of the aminosulfamoyl group attached to atom C-3 of the A-ring in compounds 1 and 2, which belong to the 5β-steroid series with a bent conformation, and compound 8, which is planar.

The active site of CA II has a narrower opening compared to CA IX due to the presence of the bulky amino acid Phe at position 130 (compared to Val in CA IX). This obstacle results in some compounds having a different binding mode—one that avoids collisions with the bulky phenylalanine side chain. Whereas compounds 7 and 8 bind in an extended conformation similar to that found in the CA IX active site, compounds 3 and 4 are rotated 180° around the bond of the sulfamoyl oxygen to C-3 of the A-ring. In this position, the C-ring interacts with residue Phe130, with carbon atom C-12 being the closest atom to Cα of Phe130. In one case, that of compound 2, the side chain Phe-130 also adjusts its position by changing the rotamer position, and the whole steroidal skeleton moves into the newly formed binding pocket. The most varied binding mode is observed with compound 5, the dihedral angle (S–O–C3–C4) is −115° when bound to CA IX active site whereas it attains a value of −82° when bound to CA II. This change brings the steroidal backbone into interaction with position 130, which is occupied by Phe in CA II and Val in CA IX (see salmon-coloured sticks in Fig. 4B).

### Structural analysis and comparison

When examining the crystal structures, we focused on the binding modes of the inhibitor compounds within the enzyme active site. By superimposing the structures and comparing key interactions, we gained insight into the structural basis for the affinity and selectivity of the inhibitor compounds towards the CA II and CA IX isoforms.

**Fig. 5:**
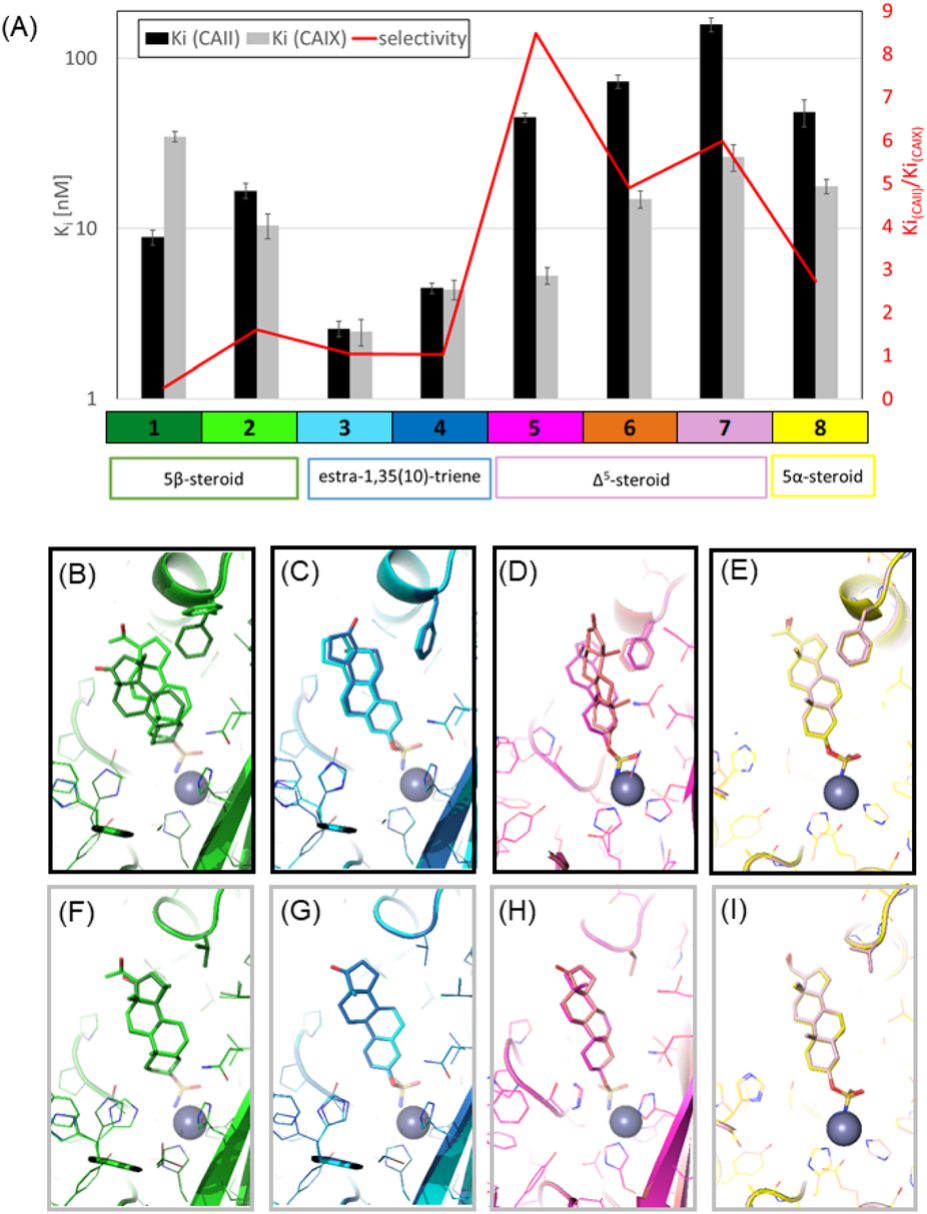
(A) The plot summarises the *K*_i_ values and selectivity of the steroid inhibitors towards CA II and CA IX, *y* axis is in logarithmic scale. (B–E) Co-crystal structures of the steroidal inhibitors bound to CA II. (F–I) Co-crystal structures of the steroidal inhibitors bound to CA IX mimic. In these structures, the inhibitor compounds are depicted as sticks, the protein as a main chain band ribbon and the key residues as sticks. The colour scheme used for the carbon atoms in each structure is explained at the bottom of the plot in panel A.

Compounds 1 and 2, both 5β-steroids, adopt a bent conformation that facilitates efficient interaction with the enzyme active site (Fig. 5B and F). The difference between these two compounds lies in the substitutions at positions C-15 and C-17 of the D-ring. The methyl group at C-15 greatly affects the binding mode whereas the C-17 modification has no apparent effect on the binding mode, as this group projects away from the active site and does not interact with any residues. Notably, compound 2 retains identical binding modes when in complex with both enzymes, while compound 1 exhibits distinct binding modes when bound to the two isoforms. The change in binding mode of 1 with CA II is caused by the combination of the methyl substitution of the compound and the presence of a bulky Phe 130 residue located at the entrance of the CA II active site. To avoid steric clashes of its methyl substituent and with the Phe side-chain, compound 1 takes on a high-affinity position, achieving a *K*_i_ of 9 nM. By contrast, the binding of compound 2 to CA II triggers a rearrangement of the Phe 130 side-chain, shifting its rotameric position to accommodate the compound (Fig. 5B). This rearrangement allows 2 to retain the same binding mode found in CA IX but results in a 2-fold lower affinity compared to 1. When in complex with CA IX, both 5β-steroid compounds have an analogous binding mode, as the hydrophobic pocket in the CA IX active site is large enough to accommodate the methyl substitution (Phe in CA II is substituted by Val in CA IX). However, compound 1 shows less than one-third the affinity of 2, and its crystal structure does not provide an explanation for this observation.

The estra-1,3,5(10)-triene compounds 3 and 4 are highly potent inhibitors of both CA II and CA IX with low nanomolar inhibition constants (Table 2). Their planar shape, which is due to the aromatic nature of the sulfonamide proximal ring A, distinguishes them within this series. They fit well into the active sites of both CA II and CA IX but exhibit different binding modes with each enzyme due to variations in the torsion angle between the sulfamate moiety and the steroid skeleton. This results in a conserved sulphonamide position but a 180° rotation of the steroid skeleton (Fig. 5C and G). Despite these differences, both binding modes form numerous van der Waals interactions with the active sites of CA II and CA IX, leading to high affinity without selectivity towards any particular active site.

Steroids of the Δ5 series, represented by compounds 5, 6 and 7, show greater selectivity towards CA IX, binding to its active site in a nearly identical manner (Fig. 5H). Comparison of the binding modes and interactions of these three compounds with the active site of CA IX does not clarify why compound 5 exhibits the highest affinity. We could speculate that the gain in affinity is due to a reduced desolvation penalty, as its hydrophobic nature contrasts with the polar substituents of compounds 6 and 7, which remain exposed to the solvent upon binding to the active site. With CA II, compounds 5 and 7 also exhibit similar binding modes. However, the presence of two alternative conformations of Phe 130 represent an adjustment in the protein active site to avoid steric clashes with the rigid steroidal backbone, which likely reduces the binding affinity of these compounds. In the case of compound 6, steric hindrances lead to a significant rearrangement of its binding mode within the CA II active site.

Differences between Δ5 and 5α-steroids, which have a different chirality at carbon C-3 and a reduced bond in the B-ring, lead to a subtle shift in the position of the sulfamide moiety and the linker between the sulfamide and steroidal moieties (Fig. 5E and I). Nonetheless, their overall binding mode remains similar, and their binding affinities are not exceptional.

## Conclusion

Our investigation into the inhibitory activity and binding modes of steroid-based compounds within the active sites of CA II and CA IX has provided a detailed understanding of their structure–activity relationships. This analysis highlights how specific structural features influence both potency and isoform selectivity, offering valuable insights into the distinct properties of the active sites of CA II and CA IX. By mapping key interactions and identifying functional groups and modifications that enhance binding, these findings can inform the rational design of next-generation inhibitors with improved affinity and selectivity, particularly toward the cancer-associated isoform CA IX, as well as other clinically relevant carbonic anhydrase isoforms.

We examined compounds representing four steroidal scaffolds, namely 5β-steroid, estra-1,3,5(10)-triene, Δ5-steroid and 5α-steroid, to assess their binding affinity and selectivity across CA isoforms. Our novel series of steroidal sulfamates (compounds 1–8) demonstrated potent nanomolar inhibition of both CA II and CA IX. Among them, estrone-based derivatives 3 and 4 emerged as the most active dual inhibitors, exhibiting *K*_i_ values of 2.6 nM and 4.5 nM against CA IX, respectively, with similarly high affinity for CA II (selectivity index ≈ 1.0). These compounds align with the broader class of steroidal sulfamates such as STX140 and its analogues, known for their dual CA inhibition and anticancer potential. A critical challenge in CA inhibitor development is achieving isoform selectivity to minimize off-target effects, particularly inhibition of ubiquitously expressed CA II, which can lead to systemic side effects. The selectivity indices of our compounds range from 0.3 to 8.5 (CA II *K*_i_/CA IX *K*_i_). The highest selectivity achieved within this study was identified for the Δ5-steroid derivative 5 with selectivity toward CA IX (*K*_i_ = 5.2 nM; selectivity index = 8.5), highlighting its promise as a candidate for further structural modifications. The reason for this conclusion is the nonaromatic nature of the molecule. Aromatic estranes are more biologically active but can indeed exhibit higher potential for toxicity compared with non-aromatic analogues, primarily because of their enhanced nuclear receptor binding and metabolic stability.

Inhibition and structural studies have demonstrated that the active sites of human carbonic anhydrase are generally able to accommodate steroidal compounds functionalized with a zinc-coordinating sulfamate group. The binding mode of these compounds varies depending on differences between isoforms of the enzyme, particularly in the hydrophobic patch at the entrance to its active site. This region is formed by conserved residues (Leu197, Val134 and Leu140) and a key variable residue at position 131 (Phe in CA II and Val in CA IX), which plays a significant role in isoform-specific interactions (Fig. 5).

Notably, derivatives of estra-1,3,5(10)-triene (compounds 3 and 4) emerged as potent nanomolar inhibitors of both CA II and CA IX. Their ability to adopt two distinct binding orientations, driven by variation in the torsion angle between the sulfamate group and the steroidal core, underpins their high affinity and lack of isoform selectivity. This structural adaptability is further supported by their strong inhibition of CA VII (Table 2).

By contrast, the Δ5- and 5α-steroid derivatives demonstrated reduced flexibility in the narrower active site of CA II, resulting in increased selectivity towards CA IX. Among these, Δ5-steroid compound 5 exhibit the greatest selectivity within the series (Table 2, and Fig. 5A), likely due to its favourable fit in the CA IX binding pocket.

In summary, steroidal frameworks represent versatile and promising scaffolds for the design of isoform-selective carbonic anhydrase inhibitors. Our results indicate that estra-1,3,5(10)-triene and Δ5-steroid derivatives, particularly those lacking substitution at C-17 (*e.g.*, compounds 3 and 5), exhibit notable inhibitory activity toward several CA isoforms. While no compounds demonstrated selectivity for CA VII in the present series, compound 3 showed potent nanomolar inhibition for both CA II and CA IX, and compound 5 displayed marked selectivity for CA IX. These findings provide valuable structure–activity insights that can guide the rational optimization of steroid-based inhibitors toward enhanced isoform specificity.

## Experimental

### Chemistry

The melting points of compounds were determined using a micromelting point apparatus (Hund/Wetzlar, Germany) and are uncorrected. Reactions were monitored by thin-layer chromatography (TLC) on Kieselgel-G (Si 254 F, Merck KGaA, Darmstadt, Germany) layers (0.25 mm thick). Spots were detected by spraying with 5% phosphomolybdic acid in absolute ethanol. *R*_f_ values were determined for spots observed under illumination with a wavelength of 254 and 365 nm. Merck silica gel 60, 40–63 µm (Merck KGaA, Darmstadt, Germany) was used for flash chromatography. Elementary analysis data were acquired using a PE 2400 Series II CHNS/O Analyzer (PerkinElmer Inc., Waltham, MA, USA) and an MX5 microbalance (Mettler Toledo, Switzerland) with ±0.40% of the calculated values confirming a purity of >95%. Optical rotation was measured using an AUTOPOL IV automatic polarimeter (Rudolph Research Analytical, NJ, USA); all samples were measured at 20 °C, at a given concentration in a given solvent at 589 nm. Proton and carbon NMR spectra were measured on a Bruker AVANCE III™ 400 MHz device with chemical shifts given in parts per million (ppm *δ*, relative to the residual CDCl_3_ peak at 7.260 and 77.160 ppm for ^1^H and ^13^C nuclei, respectively). Coupling constants (*J*) are given in Hz. For the determination of multiplicities, the J-MOD pulse sequence was used. HRMS spectra were acquired on an LTQ Orbitrap XL mass spectrometer (Thermo Fisher Scientific, Massachusetts, USA) in ESI mode. IR spectra were recorded on a Bruker IFS 55 spectrometer with wavenumbers expressed in cm^−1^. All solvents were distilled prior to use.

### General procedure for the preparation of 3-sulfamoyl derivatives

The corresponding steroid alcohol (1 eq.) was dissolved in dimethylacetamide (2.5 mL) and cooled to 0 °C, then sulfamoyl chloride (3.5 eq.) was added in one portion. The resulting solution was stirred under nitrogen and allowed to reach room temperature overnight. The conversion of the reaction was monitored by TLC; full conversion was detected after 16 h. The reaction mixture was diluted with EtOAc and washed with water (3 × 50 mL) and brine (1 × 50 mL). The aqueous phase was extracted with EtOAc (2 × 30 mL). The combined organic phase was dried over Na_2_SO_4_, filtered and concentrated *in vacuo*.

17-Oxo-15β-methyl-5β-androstan-3α-yl sulfamate (compound 1) was prepared according to the general procedure for the preparation of 3-sulfamoyl derivatives. Compound 1 was prepared with compound 12 (0.62 mmol; 190 mg) as the starting material. The crude material was purified by flash column chromatography on silica gel, using gradient elution with 10–100% EtOAc/DCM. Crystallization of the previously purified material with diethyl ether afforded compound 6 (124 mg) as a white powder with a yield of 52%. Mp: 162–164 °C, *R*_f_ = 0.70 (benzene/acetone/hexane, 1 : 1:1). [*α*]^20^_D_ + 62.1 (*c* 1.0, DMSO). ^1^H NMR (CDCl_3_, 400 MHz): *δ* 0.99 (s, 3H, H-18), 1.00 (s, 3H, H-19), 1.10 (d, 3H, *J* = 7.3 Hz, H-15), 4.56 (tt, 1H, *J*_1_ = 11.4, *J*_2_ = 4.9, H-3), 4.89 (s, 2H, NH_2_). ^13^C NMR (CDCl_3_, 101 MHz): *δ* 222.0 (C-17), 83.8 (C-3), 53.5, 47.3, 45.1, 42.1, 41.6, 35.3, 34.9, 34.5, 27.8, 27.4, 26.9, 25.4, 23.2, 20.1 (C-19), 18.0 (C-18), 17.0 (C-20). MS (ESI): *m*/*z* = 382 (100%, M − H). HR-MS (ESI): *m*/*z* for C_20_H_32_NO_4_S [M − H] calcd: 382.20575, found: 382.20532. IR spectrum (CHCl_3_): 3360, 3248, 3129 (NH); 2146, 1966, 1179 (SO2), 1715 (C=O); 1573 (NH_2_). Calcd for C_20_H_33_NO_4_S (383.55): C, 62.63; H, 8.67; N, 3.65%. Found: C, 62.63; H, 8.44; N, 3.50%.

20-Oxo-5β-pregnan-3α-yl sulfamate (compound 2) was prepared according to a procedure described in the literature.^42^

Estra-1,3,5(10)-trien-3-yl sulfamate (compound 3) was prepared according to the general procedure for the preparation of 3-sulfamoyl derivatives. Starting from compound 13 (1.0 mmol; 256.4 mg), compound 3 was prepared (186 mg) in 56% yield. Mp: 120–122 °C (EtOAc–hexane), *R*_f_ = 0.45 (petroleum ether/acetone, 4 : 1); [*α*]^20^_D_ + 64.3 (*c* 0.3, CHCl_3_). ^1^H NMR (CDCl_3_, 400 MHz): *δ* 0.74 (s, 3H, H-18), 2.26 (m, 2H), 2.87 (td, 2H, *J*_1_ = 5.8, *J*_2_ = 5.1, *J*_3_ = 2.9, H-6 and H-9) 7.03 (d, 1H, *J* = 2.6, H-4), 7.07 (dd, 1H, *J*_1_ = 8.5, *J*_2_ = 2.7, H-2), 7.32 (dd, 1H, *J*_1_ = 8.7, *J*_2_ = 2.7, H-1). ^13^C NMR (CDCl_3_, 101 MHz): *δ* 147.9 (C-3), 140.3, 139.2, 126.9, 121.9, 118.9, 53.7, 44.3, 41.1, 40.6, 38.8, 38.8, 29.8, 27.9, 26.7, 25.3, 20.7, 17.6 (C-18). MS (ESI): *m*/*z* = 358.1 (100%, M + Na), 693.3 (40%, 2M + Na). HR-MS (ESI): *m*/*z* for C_18_H_25_NO_3_SNa [M + Na] calcd: 358.14474, found: 358.14480. IR spectrum (CHCl_3_): 3453, 3353 (NH_2_); 1398, 1187 (SO_2_). Calcd for C_18_H_25_NO_3_S (335.1): C, 64.45; H, 7.51; N, 4.18%. Found: C, 64.25; H, 7.44; N, 4.41%.

17-Oxo-estra-1,3,5(10)-trien-3-yl sulfamate (compound 4) was prepared according to the procedure described in the literature.^43^

Androst-5-en-3β-yl sulfamate (compound 5) was prepared according to the general procedure for the preparation of 3-sulfamoyl derivatives with 3β-hydroxy-androst-5-ene (1.04 mmol; 300 mg) as the starting material. The crude material was purified by flash column chromatography on silica gel, using gradient elution with 10–100% EtOAc/DCM. Subsequent crystallization from hexane afforded compound 5 as a white powder in 58% yield (223 mg). Mp: 161–163 °C, *R*_f_ = 0.65 (benzene/acetone/hexane, 1 : 1 : 1). [*α*]^20^_D_ −71.2 (*c* 1.0, DMSO). ^1^H NMR (DMSO-*d*6, 400 MHz): *δ* 0.70 (s, 3H, H-18), 0.98 (s, 3H, H-19), 4.21 (m, 1H, H-3), 5.38 (d, 1H, *J* = 2.8 Hz, H-6), 7.39 (broad s, 2H, NH_2_). ^13^C NMR (DMSO-*d*6, 101 MHz): *δ* 139.3 (C-5), 122.6 (C-6), 79.6 (C-3), 52.3, 49.8, 41.2, 39.8, 38.5, 38.1, 36.6, 36.1, 31.7, 31.6, 28.3, 25.2, 20.6, 20.1, 19.0 (C-19), 17.1 (C-18). MS (ESI): *m*/*z* = 352 (100%, M − H). HR-MS (ESI): *m*/*z* for C_19_H_31_NO_3_SNa [M + Na] calcd: 376.19169, found: 376.19130. IR spectrum (CHCl_3_): 3402, 3315, 3285 (NH); 3030, 1670 (C=C); 1549 (NH); 1375, 1358, 1193 (SO_2_). Calcd for C_19_H_31_NO_3_S (353.52): C, 64.55; H, 8.84; N, 3.96%. Found: C, 64.58; H, 8.72; N, 3.71%.

17-Oxo-androst-5-en-3β-yl sulfamate (compound 6) was prepared according to the general procedure for the preparation of 3-sulfamoyl derivatives, using dehydroepiandrosterone (1.04 mmol; 300 mg) as the starting material. The crude material was crystallized from acetone, affording compound 6 (185 mg) as a white powder in 48% yield. Mp: 151–153 °C, *R*_f_ = 0.65 (benzene/acetone/hexane, 1 : 1 : 1). [*α*]^20^_D_ −13.9 (*c* 1.0, DMSO). ^1^H NMR (CDCl_3_, 400 MHz): *δ* 0.80 (s, 3H, H-18), 1.00 (s, 3H, H-19), 4.21 (m, 1H, H-3), 5.42 (d, 1H, *J* = 3.5 Hz, H-6), 7.40 (broad s, 2H, NH_2_). ^13^C NMR (DMSO-*d*_6_, 101 MHz): *δ* 219.7 (C-17), 139.5 (C-5), 122.2 (C-6), 79.5 (C-3), 50.8, 49.6, 46.8, 38.4, 36.5, 36.1, 35.2, 31.1, 30.9, 30.2, 28.2, 21.4, 19.9, 18.9 (C-19), 13.2 (C-18). MS (ESI): *m*/*z* = 366 (100%, M − H). HR-MS (ESI): *m*/*z* for C_19_H_29_NO_4_SNa [M + Na] calcd: 390.17095, found: 390.17070. IR spectrum (CHCl_3_): 3367, 3344, 3262 (NH); 3036, 1699 (C=C); 1729 (C=O), 1571 (NH_2_); 1387, 1375 (SO_2_), 1179 (SO_2_). Calcd for C_19_H_29_NO_4_S (367.50): C, 62.10; H, 7.95; N, 3.81%. Found: C, 61.96; H, 7.79; N, 3.66%.

20-Oxo-pregn-5-en-3β-yl sulfamate (compound 7) was prepared according to the general procedure for the preparation of 3-sulfamoyl derivatives, with pregnenolone (0.9 mmol; 300 mg) as the starting material. The crude material was purified by flash column chromatography on silica gel, using gradient elution with 10–100% EtOAc/DCM. Subsequent crystallization from acetone:hexane (1 : 3) afforded compound 7 (274 mg) as a white powder in 73% yield. Mp: 139–141 °C, *R*_f_ = 0.61 (benzene/acetone/hexane, 1 : 1 : 1). [*α*]^20^_D_ 0 (*c* 0.4, DMSO). ^1^H NMR (CDCl_3_, 400 MHz): *δ* 0.54 (s, 3H, H-18), 0.97 (s, 3H, H-19), 2.06 (s, 3H, H-21), 4.21 (m, 1H, H-3), 5.38 (d, 1H, *J* = 5.2 Hz, H-6), 7.40 (broad s, 2H, NH_2_). ^13^C NMR (CDCl_3_, 101 MHz): *δ* 208.6 (C-20), 139.4 (C-5), 122.4 (C-6), 79.5 (C-3), 62.6, 56.0, 49.4, 43.3, 38.5, 37.9, 36.5, 36.0, 31.2 (C-21), 31.27, 31.31, 28.2, 24.0, 22.2, 20.6, 19.0 (C-19), 13.0 (C-18). MS (ESI): *m*/*z* = 394 (100%, M − H). HR-MS (ESI): *m*/*z* for C_21_H_32_NO_4_S [M − H] calcd: 394.20575, found: 394.20512. IR spectrum (CHCl_3_): 3351, 3247, 3103 (NH); 1685 (C=O); 1580, 1545 (SO_2_). Calcd for C_21_H_33_NO_4_S (395.56): C, 63.77; H, 8.41; N, 3.54%. Found: C, 62.71; H, 8.21; N, 3.39%.

20-Oxo-5α-pregnan-3α-yl sulfamate (compound 8) was prepared according to a procedure described in the literature.^42^

3α-Hydroxy-5β-androstan-17-one (compound 9) is available commercially from various sources, for example Sigma-Aldrich (CAS 53-42-9, catalogue number E5126).

3α-Hydroxy-16-(phenylsulfinyl)-5β-androstan-17-one (compound 10). Added to a solution of *t*-BuOK (5.5 g, 49 mmol) in dry THF (100 mL) was compound 9 (7.1 g, 24.5 mmol) in dry THF (20 mL) at room temperature under an inert atmosphere. After stirring at room temperature for 10 minutes, methyl benzenesulfinate (7.66 g, 49 mmol) was added. After stirring for 30 minutes at 30 °C, the reaction was quenched with water (100 mL) and extracted with EtOAc (2x). The combined organic extracts were washed with brine and dried over Na_2_SO_4_. The solvents were evaporated, and the crude material was purified by flash column chromatography on silica gel (0–100% EtOAc in hexane), affording compound 10 as a sticky solid (9.9 g), which was used for the next reaction step without further characterization. *R*_f_ = 0.28 (benzene/acetone/hexane, 1 : 1 : 1).

3α-Hydroxy-5β-androst-15-en-17-one (compound 11). To a mixture of compound 10 (9.9 g; 23.8 mmol) in xylene (200 mL) Na_2_CO_3_ (38.3 g; 0.36 mol) was added in portions. After stirring at 140 °C for 12 hours under an inert atmosphere, the mixture was diluted with EtOAc (100 mL) and washed with water (3 × 50 mL) and brine (2 × 50 mL). The combined aqueous phases were extracted with EtOAc (2 × 50 mL). Then, the combined organic phase was dried over MgSO_4_, filtered and evaporated *in vacuo*. The crude material was purified by flash column chromatography on silica gel, using gradient elution with 0–100% DCM in hexane and 0–100% EtOAc in dichloromethane, affording compound 11 (1.6 g) in the form of a pale-yellow crystalline substance (23%). *R*_f_ = 0.43 (benzene/acetone/hexane, 1 : 1 : 1). [*α*]^20^_D_ −24.3 (*c* 1.0, CHCl_3_). ^1^H NMR (CDCl_3_, 400 MHz): *δ* 0.99 (s, 3H, H-19), 1.04 (s, 3H, H-18), 3.65 (tt, *J*_1_ = 11.0 Hz, *J*_2_ = 4.7 Hz, H-3), 6.02 (dd, *J*_1_ = 6.0 Hz, *J*_2_ = 3.1 Hz, H-15), 7.51 (ddd, 1H, *J*_1_ = 6.0 Hz, *J*_2_ = 1.9 Hz, *J*_3_ = 0.8 Hz, H-16). ^13^C NMR (CDCl_3_, 125 MHz): *δ* 213.4 (C-17), 158.7 (C-15), 131.8 (C-14), 71.6 (C-3), 57.1, 51.2, 42.2, 42.1, 36.5, 35.3, 35.1, 32.9, 30.5, 29.4, 26.9, 25.5, 23.4, 20.8, 19.8. MS (ESI): *m*/*z* = 289 (70%, M + H). HR-MS (ESI): *m*/*z* for C_19_H_28_O_2_Na [M + Na] calcd: 311.19815, found: 311.19822. IR spectrum (CHCl_3_): 3609 (OH), 2977 (CH_3_); 2937 (CH_2_), 2865 (CH_2_), 1704 (C=O), 1662 (C=C). Calcd for C_19_H_28_O_2_ (288.43): C, 79.12; H, 9.79%. Found: C, 79.37; H, 9.51%.

3α-Hydroxy-15β-methyl-5β-androst-17-one (12). To a solution of MeMgBr (1.4 M in THF/toluene 1 : 3, 21 mL, 28 mmol) in dry THF (30 mL) CuI (5.3 g, 28 mmol) was added at 0 °C. After stirring at 0 °C for 30 minutes, a solution of compound 11 (1.6 g, 5.5 mmol) in dry THF (30 mL) was added. The reaction mixture was stirred at 0 °C for 3 hours. Then, the mixture was poured into a saturated solution of NH_4_Cl (50 mL) and extracted with EtOAc (3 × 30 mL). Combined organic extracts were washed with brine and dried over Na_2_SO_4_. The residue was purified by flash column chromatography using silica gel (gradient of dichloromethane, followed by 0–30% EtOAc with dichloromethane), affording compound 12 (1.0 g, 60%). Mp: 59–61 °C, *R*_f_ = 0.86 (benzene/acetone/hexane, 1 : 1 : 1). [*α*]^20^_D_ + 38.3 (*c* 1.0, CHCl_3_). ^1^H NMR (CDCl_3_, 400 MHz): 0.98 (s, 3H, H-18), 1.00 (s, 3H, H-19), 1.11 (d, 3H, *J* = 7.3 Hz, H-20), 3.64 (tt, 1H, *J*_1_ = 10.7, *J*_2_ = 4.7 Hz, H-3). ^13^C NMR (CDCl_3_, 101 MHz): *δ* 222.04 (C-17), 71.76 (C-3), 53.55, 47.35, 45.13, 42.07, 41.63, 36.59, 35.63, 35.09, 34.57, 33.19, 30.59, 27.90, 27.23, 25.60, 23.43, 20.13, 18.04, 17.07 (C-20). MS (ESI): *m*/*z* = 327 (100%, M + Na). HR-MS (ESI): *m*/*z* for C_20_H_32_O_2_Na [M + Na] calcd: 327.22945, found: 327.22953. IR spectrum (CHCl_3_): 3609 (OH); 2977, 1388, 1377 (CH_3_); 1727 (C=O). Calcd for C_20_H_32_O_2_ (304.47): C, 78.90; H, 10.59%. Found: C, 78.56; H, 10.28%.

Estra-1,3,5(10)-trien-3-yl sulfamate (13). Compound 13 was prepared by a procedure published in the literature, utilizing the Wolff–Kishner reduction of steroid ketones.^44^

3β-Hydroxy-androst-5-ene (14). Compound 14 was prepared according to a procedure described in the literature.^50^

### Protein cloning, expression and purification

The corresponding recombinant CA II, referred to as CA IX mimic (CA II containing amino acid substitutions A65S, N67Q, E69T, I91L, F130V, K169E and L203A), and CAVII, containing substitution C183S and C217S, were prepared by heterologous expression in *E. coli* and purified by procedures described in a previous study.^46^ The extracellular part of CA IX, comprising the PG and CA domains (residues 38–391) and including amino acid substitution C174S, was expressed in HEK 293 cells and purified as described in a previous study.^51^

### Carbonic anhydrase inhibition assay

Recombinant CA II expressed in *E. coli* and the extracellular part of CA IX comprising residues 38–391 (PG and CA domains) expressed in HEK 293 cells were used in inhibition assays. A stopped-flow instrument (SFM-3000 by Biologic) was used for measuring the CA-catalysed CO_2_ hydration activity in the presence of inhibitors.^52^ The assay buffer consisted of 0.2 mM phenol red (a pH indicator with an absorbance maximum at 557 nm), 20 mM HEPES–Na (pH 7.5) and 20 mM Na_2_SO_4_. The concentration of CA II in the enzyme assay was 1.0–14.0 nM, concentration of CAIX was 1–4 nM and concentration of CAVII 14 nM.

To stabilize CA IX during the measurements, 0.00125% dodecyl-β-d-maltopyranoside (DDM, Anatrace) was included in the reaction mixture. The substrate (CO_2_) concentration in the reaction was 8.5 nM. Rates of the CA-catalysed CO_2_ hydration reaction were followed for a period of 30 seconds at 25 °C. Four traces of the substrate conversion in the reaction were used to determine the rate of reaction by fitting of exponential function to measured data for each inhibitor concentration.

The uncatalysed rates were determined in the same manner and subtracted from the total observed rates. Final concentration of DMSO in reaction was below 1.0% and test confirmed that addition of DMSO has negligible effect on enzyme activity (Fig. S1, in each reaction). Apparent 
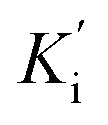
 values were obtained from dose–response curves recorded for at least six different concentrations of the test compound by the non-linear least squares method, using a spreadsheet fitting the Williams–Morrison equation.^53^*K*_i_ values were then derived using the Cheng–Prusoff equation^54^ with *K*_m_ values of 9.3 mM for CA II, 12.4 mM for CA VII and 7.5 mM for CA IX.^55^ Results of inhibition measurements are summarized in Fig. S2–S7 and were deposited in ASEP, institutional data repository of the Czech Academy of Sciences, https://doi.org/10.57680/asep.0637210.

### Protein crystallization and X-ray data collection

CA II and CA IX mimic were used for X-ray studies. Complexes of CA II and CA IX mimic with inhibitor compounds were prepared by addition of a 1.1-fold molar excess of the respective inhibitor (dissolved in pure DMSO) to a 25-mg mL^−1^ protein solution in 50 mM tris, pH 7.8. The best crystals were prepared by the hanging drop vapour diffusion method at 18 °C, using a precipitation solution containing 1.6 M sodium citrate, 50 mM Tris–HCl, pH 7.8. Drops containing 2 µL of the complex solution were mixed with 1 µL of the precipitant solution and equilibrated over a reservoir containing 1 mL of the precipitant solution. The final DMSO concentration in the drop did not exceed 5% (v/v). Crystals 0.5 × 0.3 × 0.2 mm in size typically grew within 1–4 weeks. Before data collection, the crystals were soaked for 5–10 s in a reservoir solution supplemented with 20% (v/v) sucrose and stored in liquid N_2_. Diffraction data at 100 K were collected on the BL14.1 beamline at the BESSY II electron storage ring (Berlin-Adlershof, Germany) operated by the Helmholtz-Zentrum Berlin.^56^ Diffraction data were processed using the XDS software suite.^57^ Crystal parameters and data collection statistics are summarized in Table S1 (SI).

### Structure determination, refinement and analyses

The crystal structures of CA II and CA IX mimic in complexes with steroidal inhibitor compounds were determined by the difference Fourier technique. Coordinates from PDB entries 3PO6 (ref. 58) and 6Z04 (ref. 59) were used as a model. Atomic coordinates of inhibitor molecules were generated by quantum mechanical optimizations in the Turbomole package^60^ with the density functional theory method, using the B-LYP functional and the SVP basis set, augmented with the empirical dispersion correction.^61^ The geometric library for the inhibitors was generated using the Libcheck program, which is part of the CCP4 package.^62^ The Coot^63^ program was used for inhibitor fitting, model rebuilding and the addition of water molecules. Refinement was carried out with Refmac5,^64^ with 5% of reflections reserved for cross-validation.^65^

The structures were first refined with isotropic atomic displacement parameters (ADPs). After adding solvent atoms and zinc ions, building inhibitor molecules in the active site, and exploring several alternate conformations for a number of residues, anisotropic ADPs were refined for nearly all atoms, including those in the inhibitor molecules (with the exception of spatially overlapping atoms in segments with alternate conformations; oxygen atoms of water molecules with an unrealistic ratio of ellipsoid axes were refined using isotropic ADPs). However, anisotropic ADPs were applied only to structures having a resolution better than 1.3 Å. The quality of the crystallographic model was assessed with MolProbity.^66^ The final refinement statistics are summarized in Tables S1 and S2 (SI). All structural figures were created using PyMOL.^67^ Atomic coordinates and structure factors for the crystal structures were deposited in the Protein Data Bank under accession codes 8OMP, 8OMN, 8OMH, 8OMB, 8OKQ, 8OLM, 8OLK and 8OLI (CA II complexes) and 8OKP, 8OLA, 8OLF, 8OKT, 8OKO, 8OKE, 8OKG and 8OKJ (CA IX mimic complexes).

## Author contributions

AK, VK, BS synthetized and characterized the steroidal inhibitor compounds; IS prepared and characterized enzymes, KP performed enzyme inhibition studies and crystallized proteins, and JB performed X-ray structural analysis. PR and EK designed the study, interpreted the data and wrote the manuscript. All authors have provided critical feedback and approved the final manuscript.

## Conflicts of interest

There are no conflicts to declare.

## Supplementary Material

RA-016-D5RA06507K-s001

## Data Availability

The data supporting this article have been included as part of the supplementary information (SI). Supplementary information is available. See DOI: https://doi.org/10.1039/d5ra06507k. Atomic coordinates and structure factors for the crystal structures have been deposited in the Protein Data Bank (PDB codes: 8OMP, 8OMN, 8OMH, 8OMB, 8OKQ, 8OLM, 8OLK, 8OLI, 8OKP, 8OLA, 8OLF, 8OKT, 8OKO, 8OKE, 8OKG and 8OKJ). Results of inhibition measurements were deposited in ASEP, institutional data repository of the Czech Academy of Sciences.^68*a*–*q*^
