# Supplementary material for: Steroidal A/B-ring fusion as a strategy for isoform-selective inhibition of human carbonic anhydrases
Source: RSC Adv. 2026 Feb 18;16(11):9743–55. doi: 10.1039/d5ra06507k (PMC12914371; doi:10.1039/d5ra06507k)
Supplement: RA-016-D5RA06507K-s001 [file RA-016-D5RA06507K-s001.pdf]

Supplementary Information (SI) for RSC Advances.
This journal is © The Royal Society of Chemistry 2026

Supporting Information

Steroidal A/B-Ring Fusion as a Strategy for Isoform-Selective Inhibition of Human Carbonic Anhydrases

Jiří Brynda,[a] Anita Kiss,[a] Klára Pospíšilová,[a] Vojtěch Kapras,[a] Irena Sieglová,[a] Barbora Slavíková,[a] Pavlína Řezáčová*[a], Eva Kudová*[a]

[a]Institute of Organic Chemistry and Biochemistry of the Czech Academy of Sciences, Flemingovo namesti 2, 16000 Prague, Czech Republic

Table of Contents

^{1}H and ^{13}C NMR spectra of compounds 1,3,5-7 2

HR-MS spectra of compounds 1,3,5-7 7

Effect of DMSO on enzyme activity 10

Inhibition of CA II 11

Inhibition of CA IX 13

Inhibition of CA VII 15

Structure of inhibitors bound to the active site of CA II 17

Structure of inhibitors bound to the active site of CA IX-mimic 18

Table S1. Diffraction data collection and refinement statistics of the CAII complexes 19

Table S2. Diffraction data collection and refinement statistics of the CAIX-mimic complexes 20

Supplementary references 21

^{1}H and ^{13}C NMR spectra of compounds 1,3,5-7

^{1}H and ^{13}C NMR spectrum ($CDCl_3$, 400 MHz and 101 MHz) of compound 1

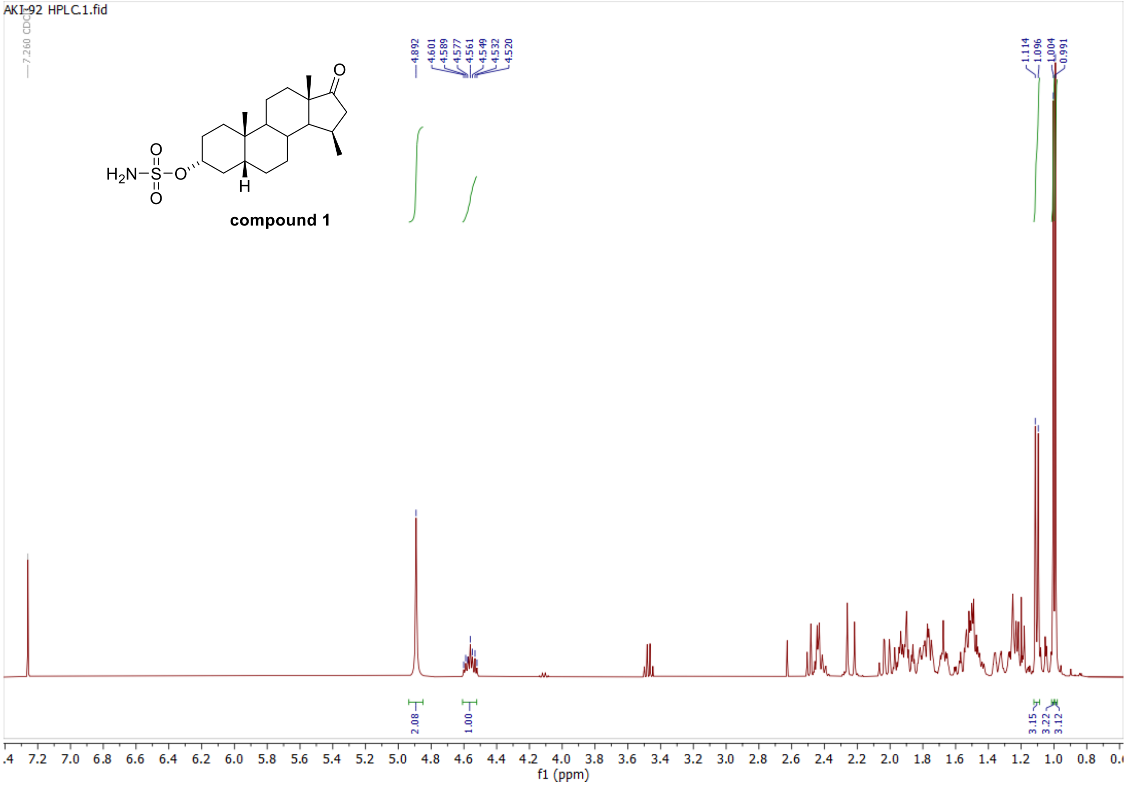

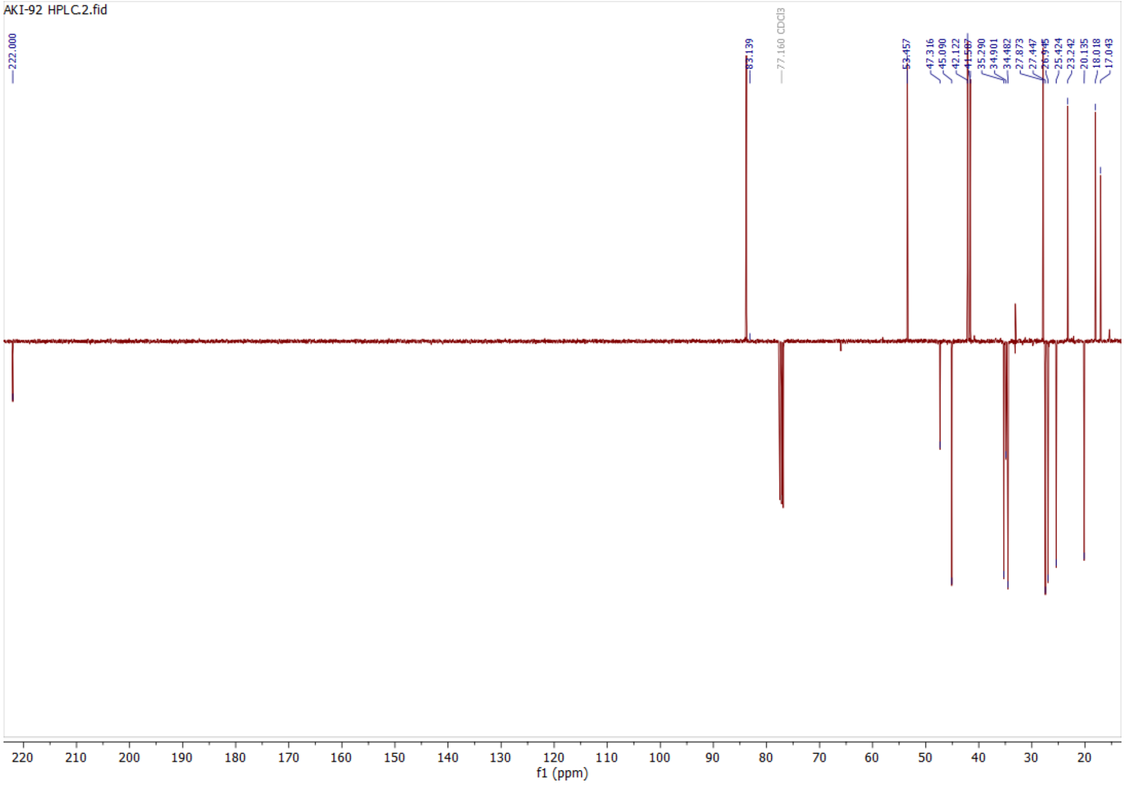

^{1}H and ^{13}C NMR spectrum ($CDCl_6$, 400 MHz and 101 MHz) of 3

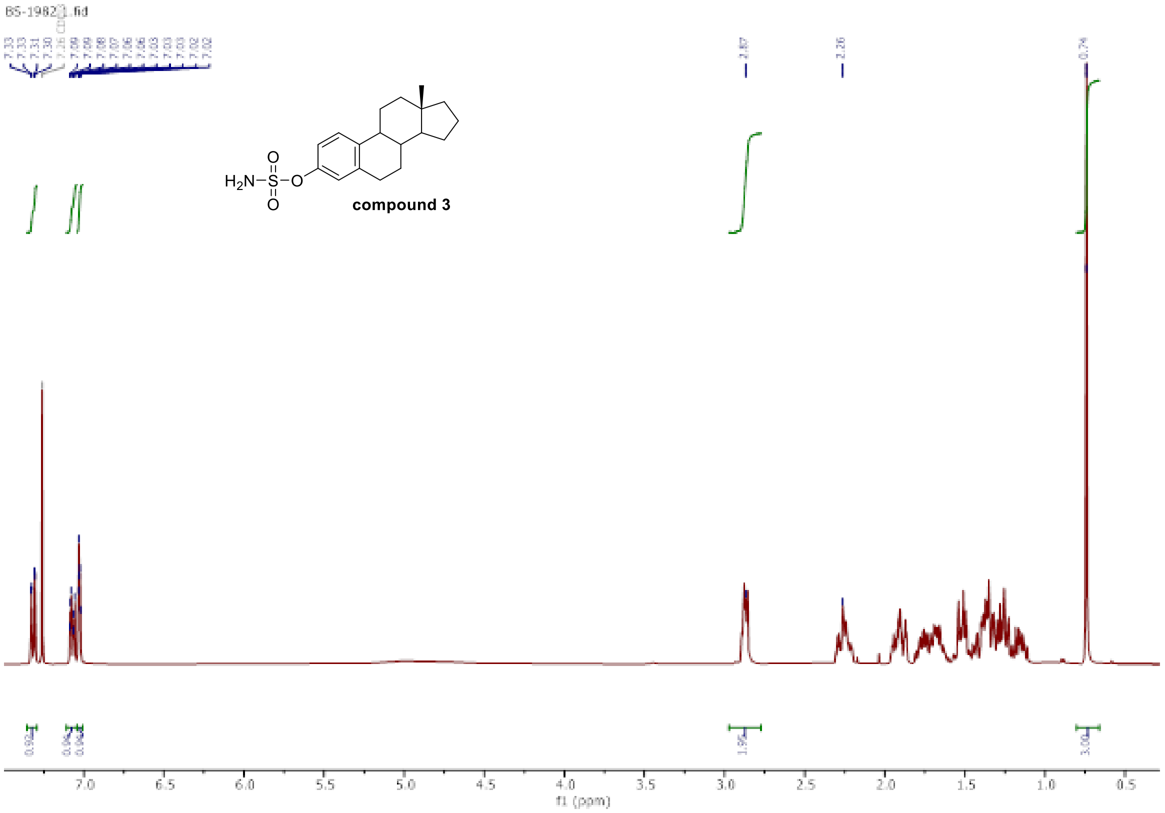

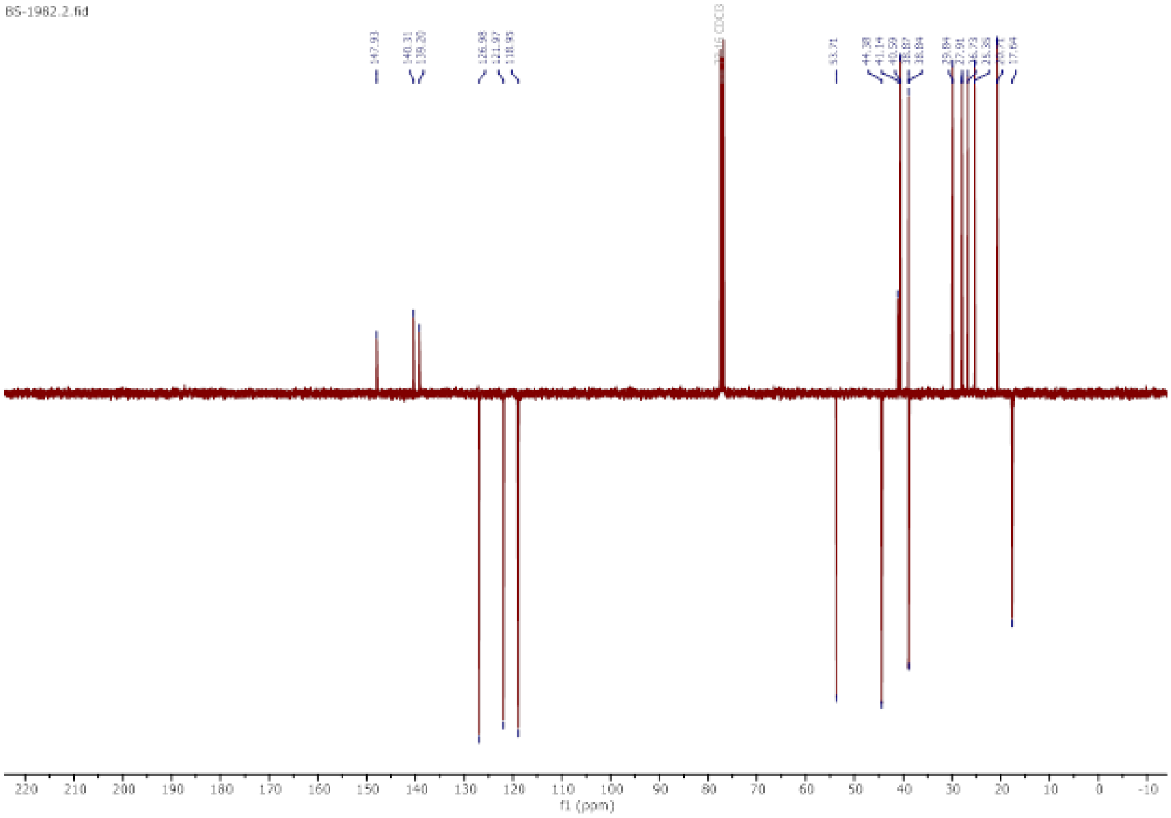

^{1}H and ^{13}C NMR spectrum (DMSO-d_6, 400 MHz and 101 MHz) of 5

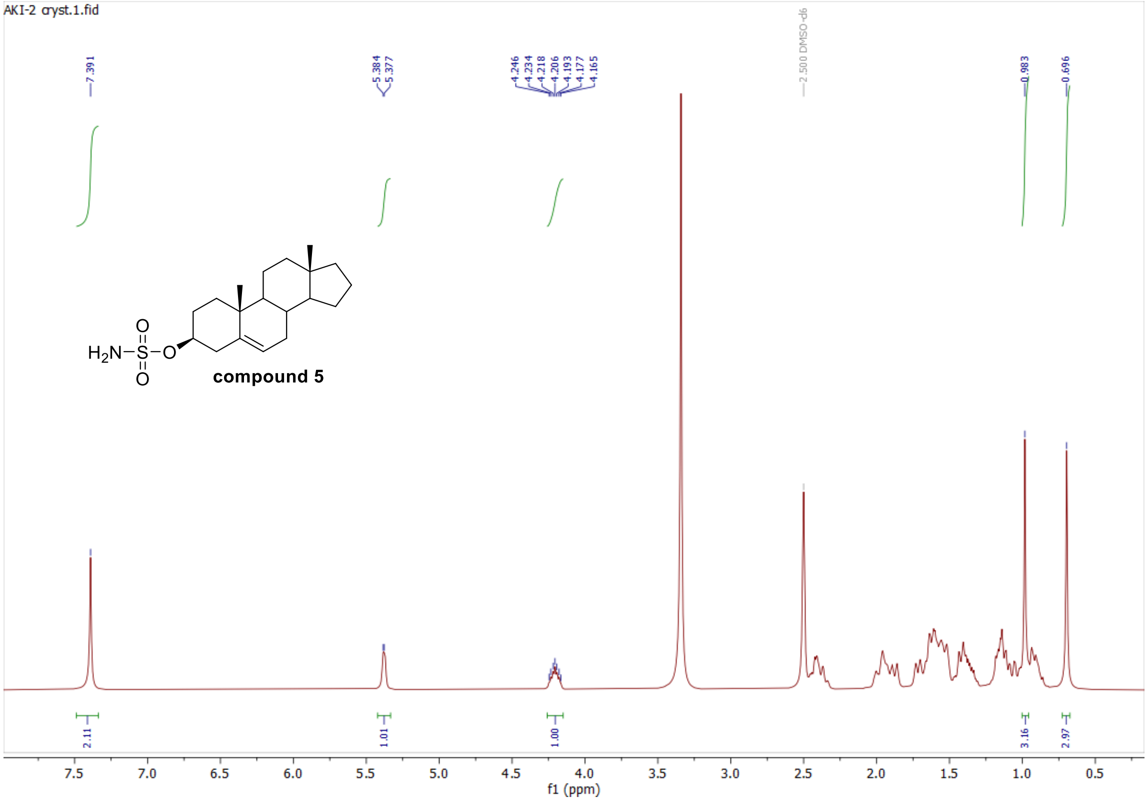

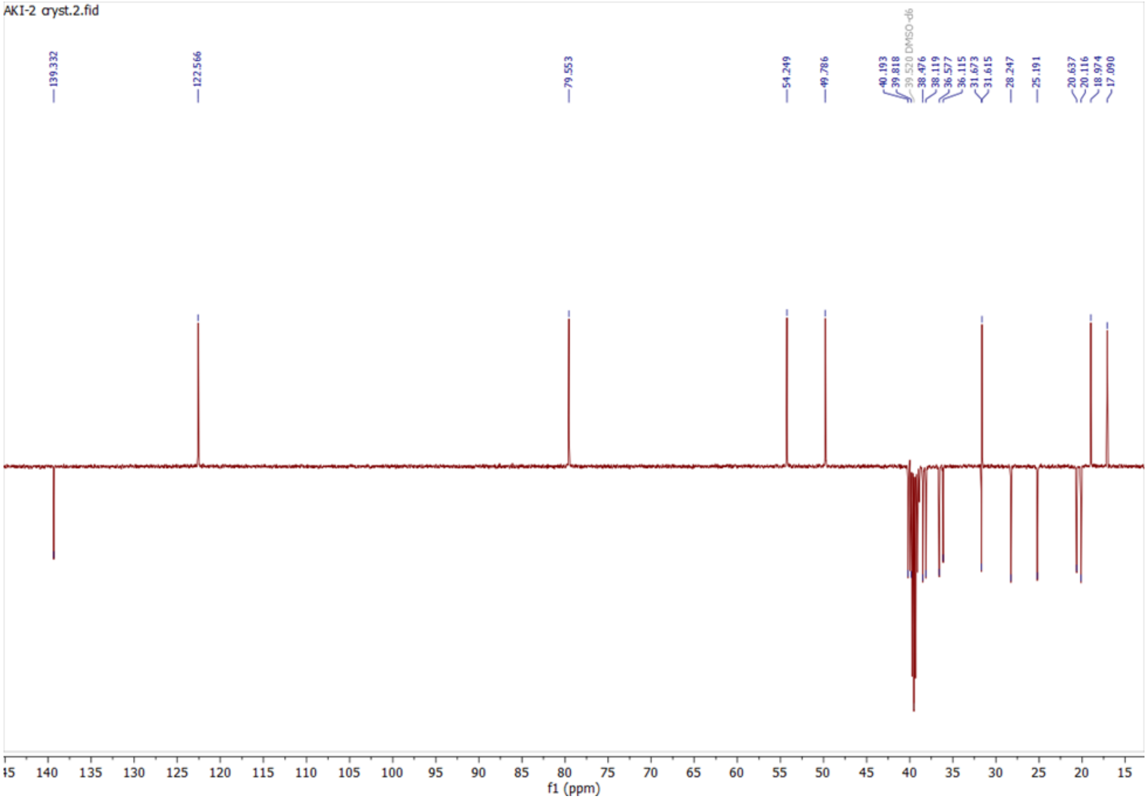

^{1}H and ^{13}C NMR spectrum (DMSO-d_6, 400 MHz and 101 MHz) of compound 6

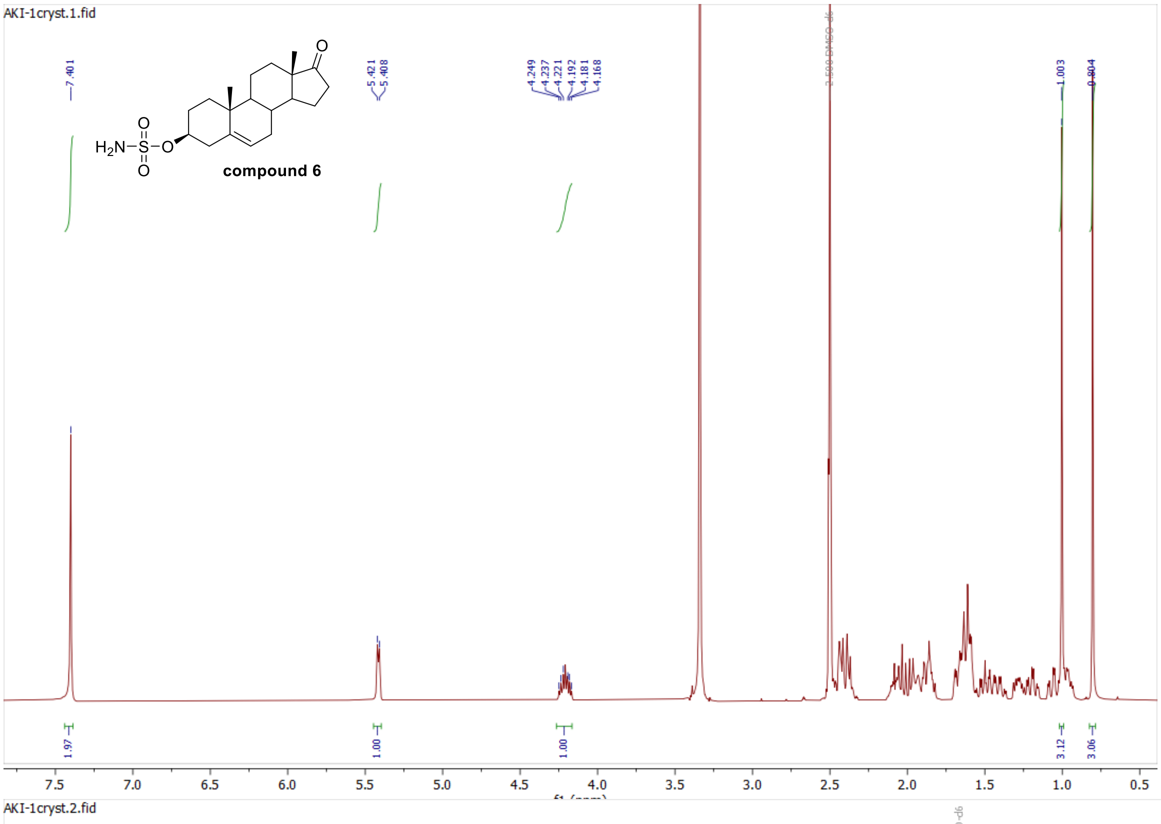

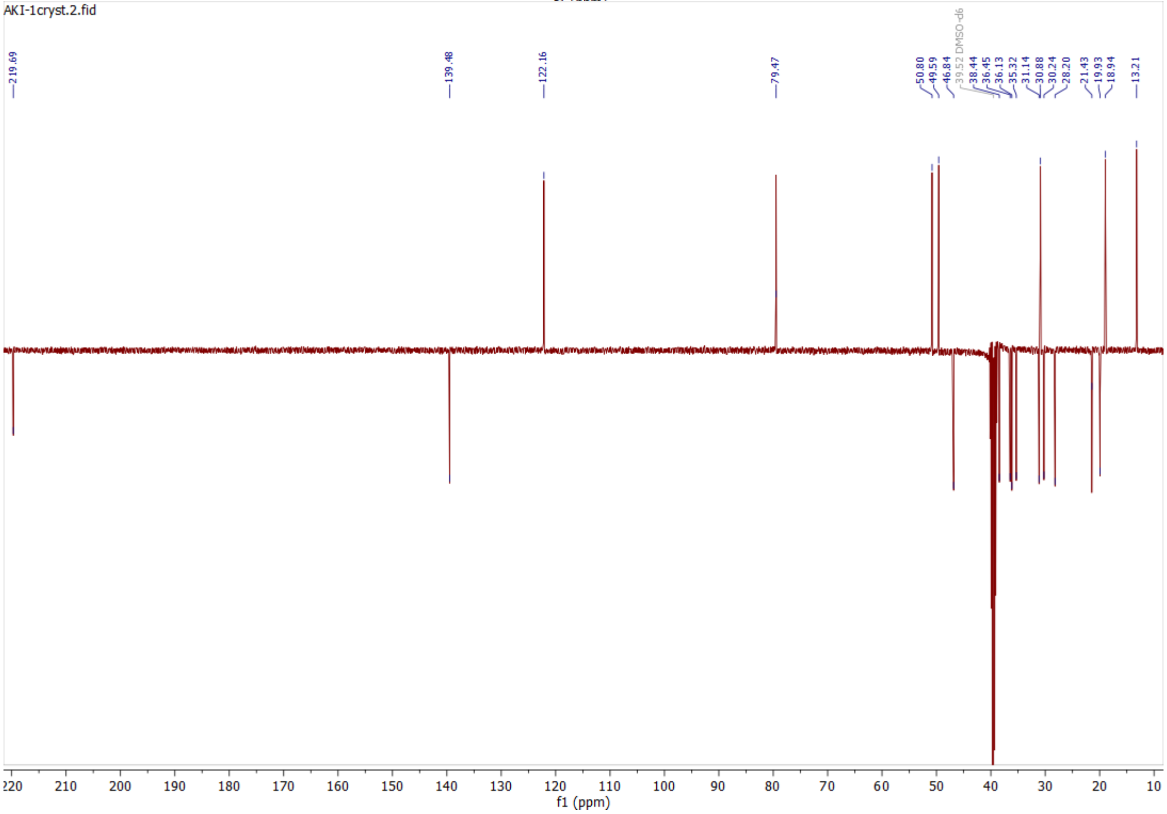

^{1}H NMR (600 MHz, DMSO-*d*$_6$) and ^{13}C NMR spectrum (150.9 MHz, DMSO-*d*$_6$) of compound **7**

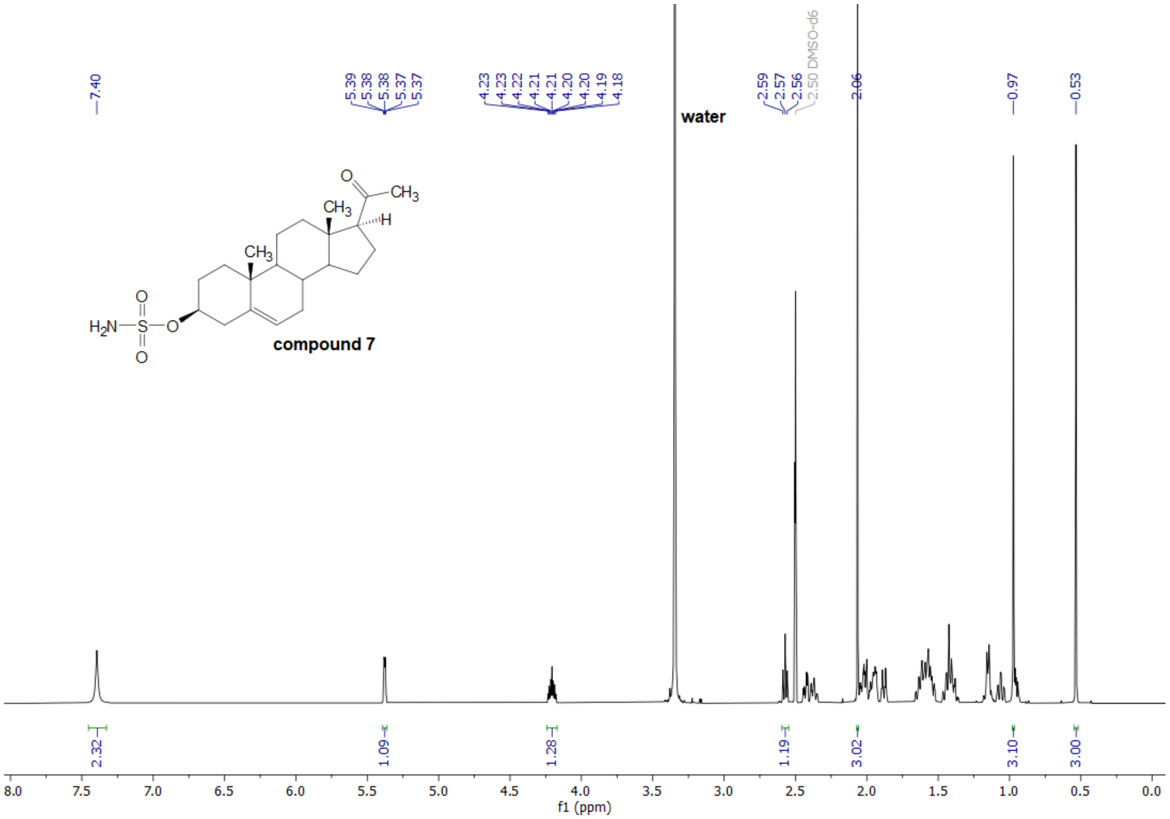

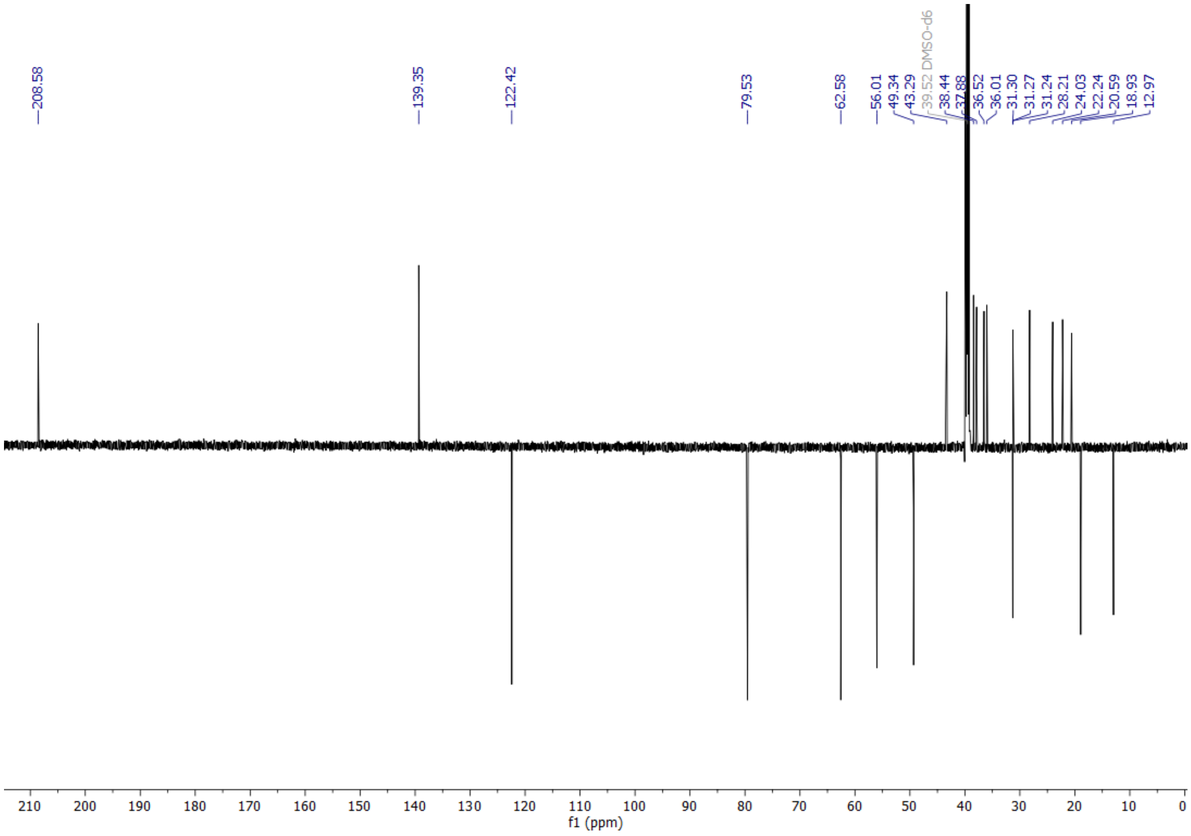

HR-MS spectra of compounds 1,3,5-7

HR-MS (ESI pos.) of compound 1

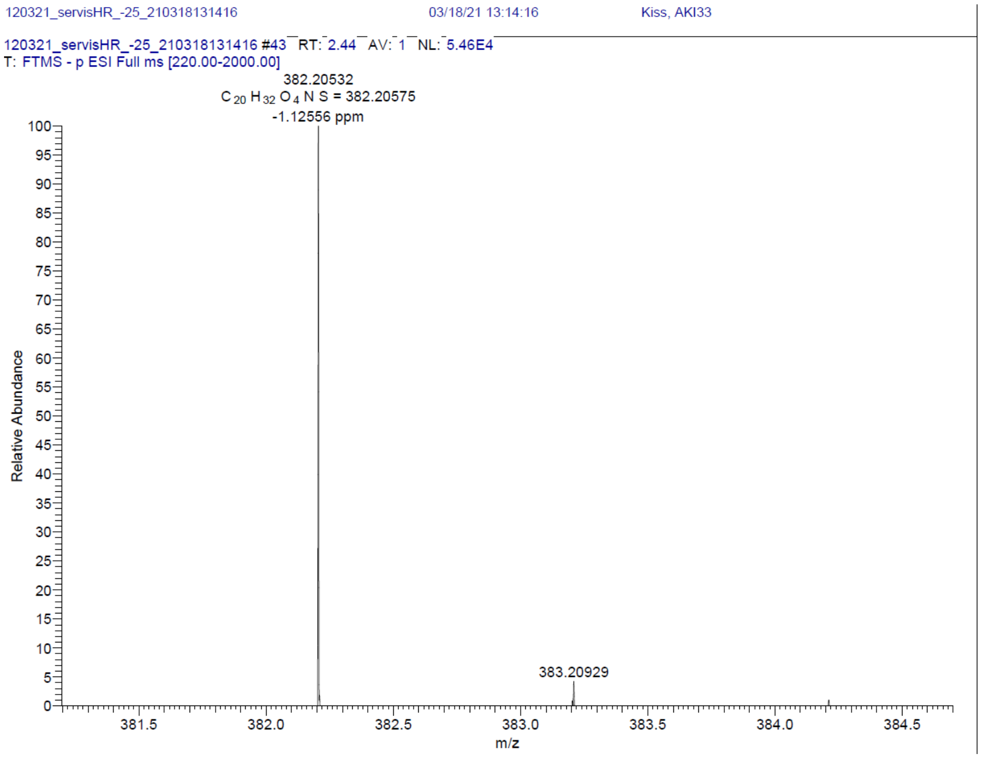

HR-MS (ESI pos.) of compound 3

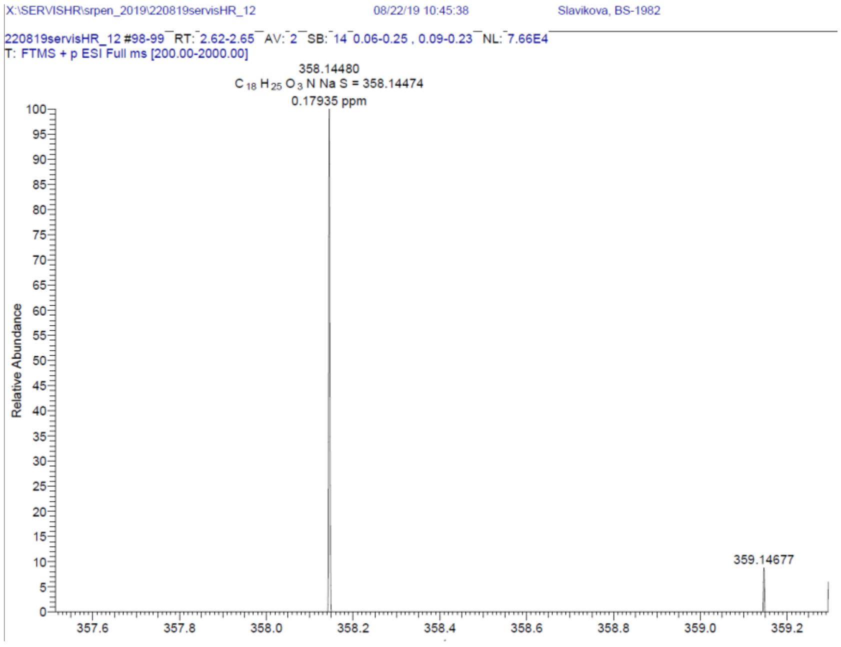

HR-MS (ESI pos.) of compound 5

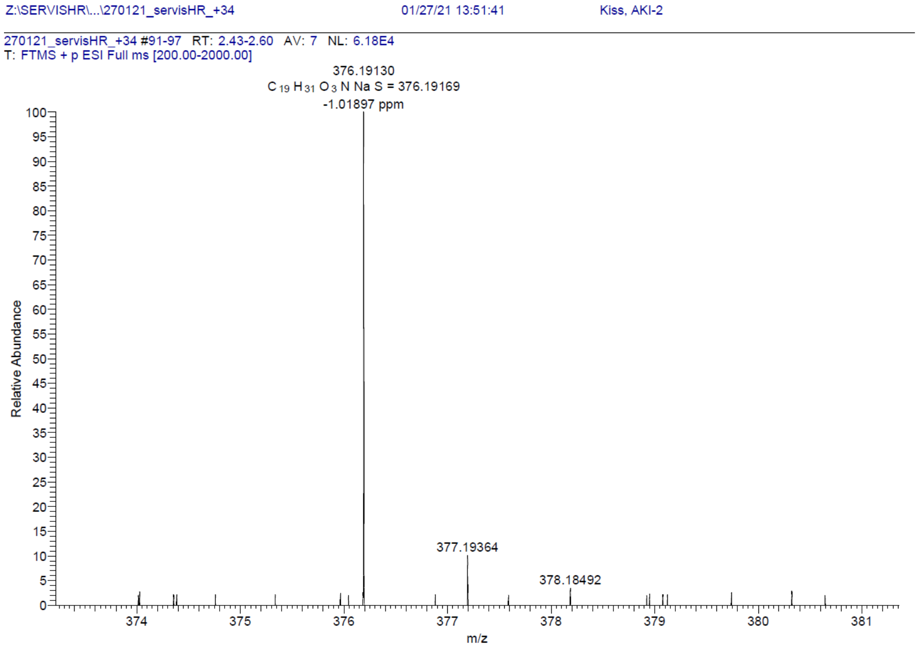

HR-MS (ESI pos.) of compound 6

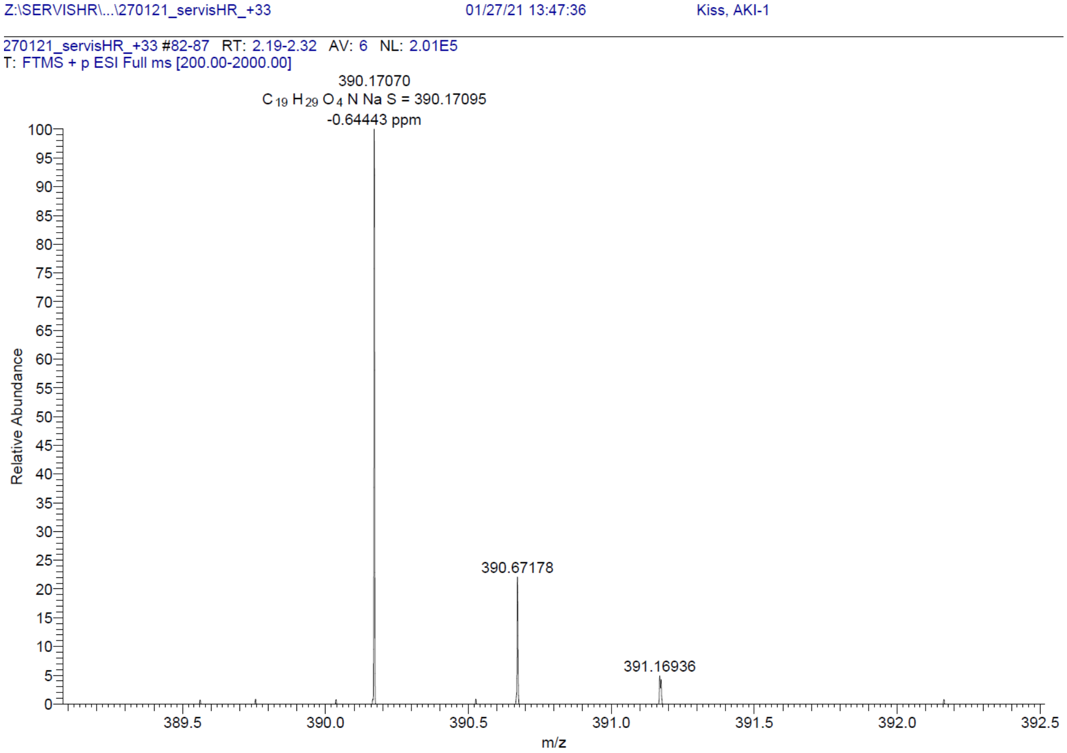

HR-MS (ESI pos.) of compound 7

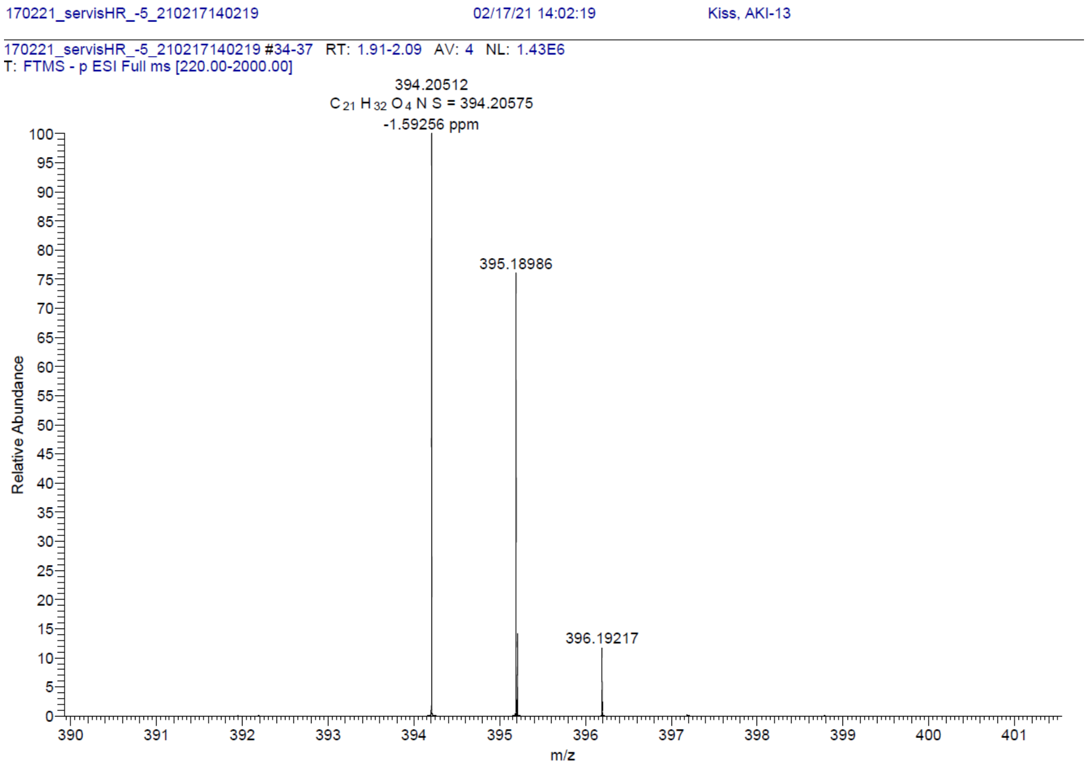

Effect of DMSO on enzyme activity

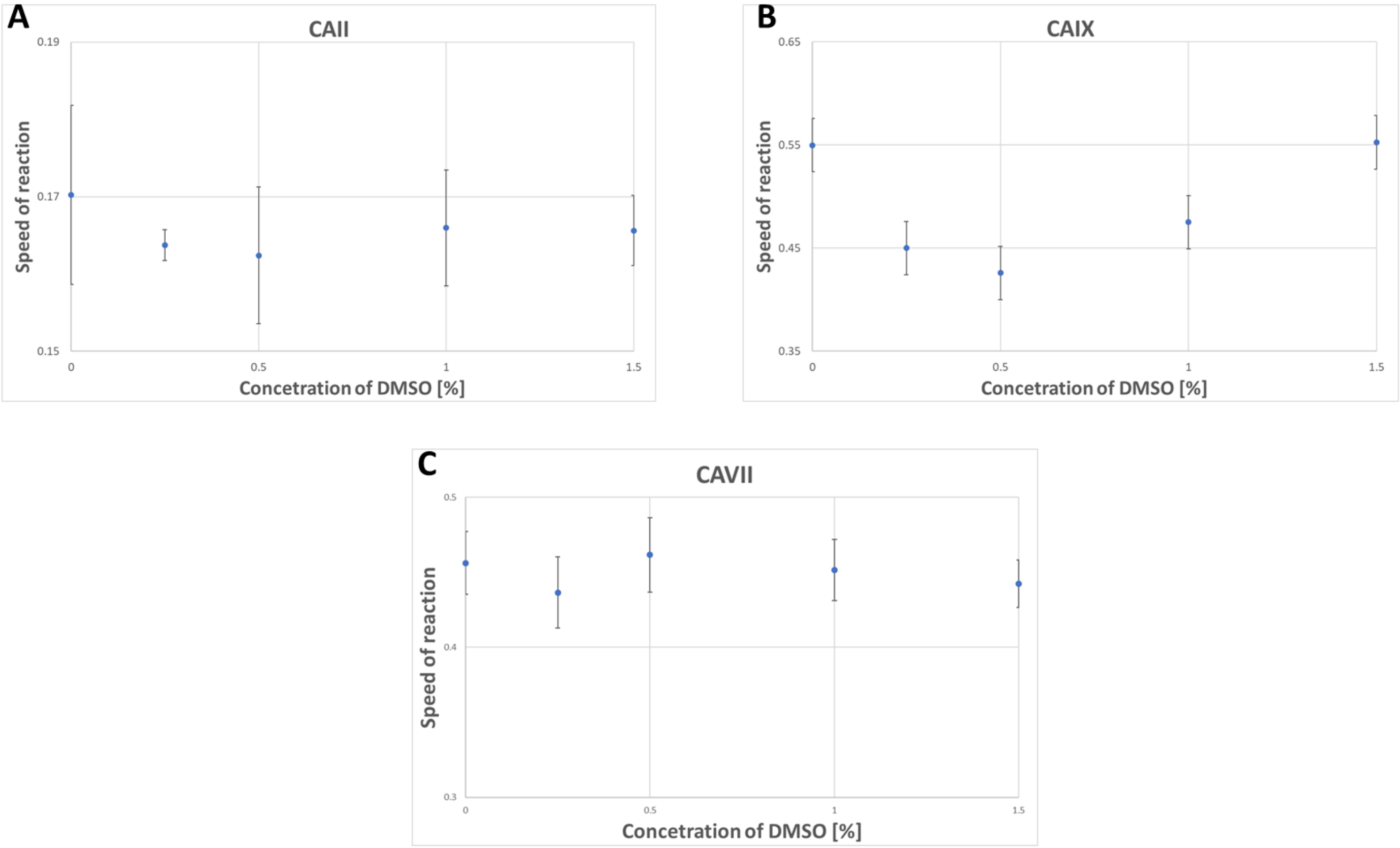

Figure S1: Activity of CAII (A), CAIX (B) and CAVII (A) enzymes in the presence of DMSO was explored by following the speed of reaction.

Inhibition of CA II

CAII inhibition

1

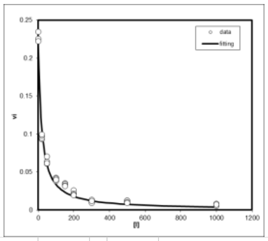

[E] =	4	±	4.879	* 1E-9 M
v_0 =	0.224	±	0.004	* 1E+0 mol.s-1
n =	36			
**K_i =**	**8.2**	**±**	**0.8**	*** 1E-9 M**

2

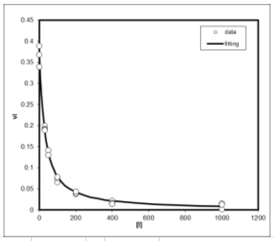

[E] =	14	±	6.034	* 1E-9 M
v_0 =	0.367	±	0.005	* 1E+0 mol.s-1
n =	25			
**K_i =**	**16.74**	**±**	**1.68**	*** 1E-9 M**

3

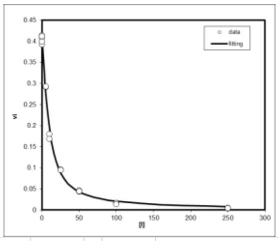

[E] =	8.972	±	1.313	* 1E-9 M
v_0 =	0.407	±	0.004	* 1E+0 mol.s-1
n =	23			
**K_i =**	**2.58**	**±**	**0.268**	*** 1E-9 M**

4

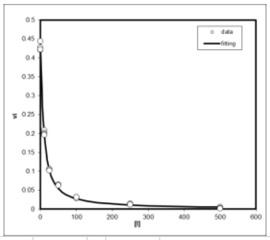

[E] =	6	±	1.211	* 1E-9 M
v_0 =	0.428	±	0.003	* 1E+0 mol.s-1
n =	25			
**K_i =**	**4.468**	**±**	**0.315**	*** 1E-9 M**

5

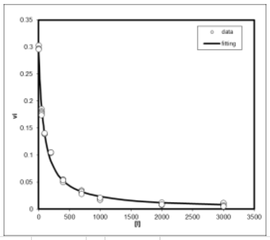

[E] =	4	±	12.02	* 1E-9 M
v_0 =	0.295	±	0.004	* 1E+0 mol.s-1
n =	36			
**K_i =**	**44.87**	**±**	**2.811**	*** 1E-9 M**

6

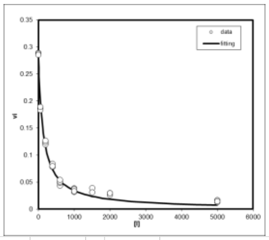

[E] =	4	±	30.82	* 1E-9 M
v_0 =	0.279	±	0.005	* 1E+0 mol.s-1
n =	34			
**K_i =**	**73.27**	**±**	**6.501**	*** 1E-9 M**

7

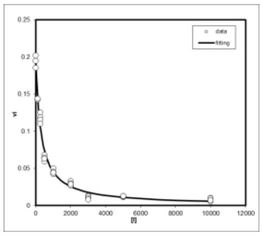

[E] =	14	±	64.58	* 1E-9 M
v_0 =	0.194	±	0.004	* 1E+0 mol.s-1
n =	34			
**K_i =**	**157.6**	**±**	**14.48**	*** 1E-9 M**

8

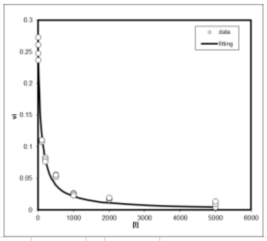

[E] =	4	±	43.51	* 1E-9 M
v_0 =	0.253	±	0.005	* 1E+0 mol.s-1
n =	24			
**K_i =**	**48.37**	**±**	**8.509**	*** 1E-9 M**

AZA

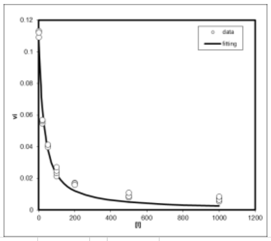

[E] =	10	±	9.852	* 1E-9 M
v_0 =	0.108	±	0.003	* 1E+0 mol.s-1
n =	26			
**K_i =**	**12.59**	**±**	**2.006**	*** 1E-9 M**

U-104

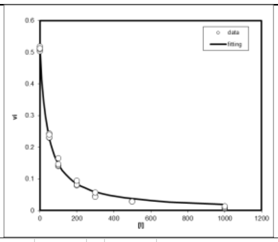

[E] =	4	±	7.913	* 1E-9 M
v_0 =	0.512	±	0.005	* 1E+0 mol.s-1
n =	27			
**K_i =**	**20.36**	**±**	**1.425**	*** 1E-9 M**

Figure S2: Inhibition of CA II by rising concentration of compounds **1-8**. Plots show the dose dependent decrease of catalytic activity expressed as speed of decrease in absorbance (v_i expressed in $\Delta A.s^{-1}$). The concentration of compound [I] in shown in $nmol.l^{-1}$. Each circle represents one measurement, black line shows fitted curves by the nonlinear least-squares method using Williams-Morrison equation. Enzyme concentration [E], initial velocity v_0, number of measurements (n) and K_i value are shown below each plot. Two know inhibitors of CAs were uses as controls:

AZA	2
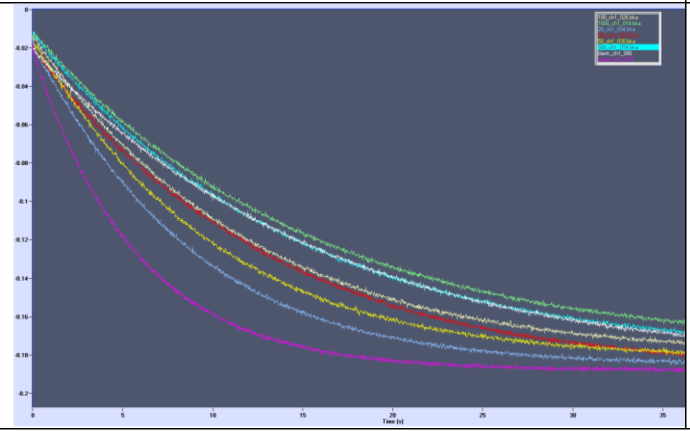	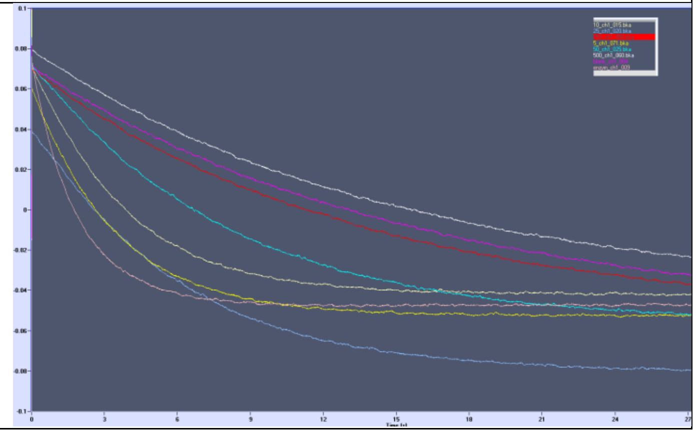

Figure S3: Raw data curves from stopped flow activity assay for CAII isoenzyme. The absorbance decrease as a function of time in reactions containing increasing concentrations of inhibitors: compound acetacolamide (AZA) and compound **2.**

Inhibition of CA IX

CAIX inhibition

1

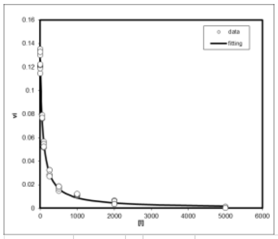

[E] =	1	±	12.85	* 1E-9 M
v_0 =	0.129	±	0.001	* 1E+0 mol.s-1
n =	34			
**K_i =**	**34.91**	**±**	**2.489**	*** 1E-9 M**

2

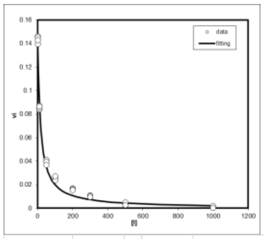

[E] =	1	±	3.655	* 1E-9 M
v_0 =	0.14	±	0.002	* 1E+0 mol.s-1
n =	27			
**K_i =**	**10.46**	**±**	**1.096**	*** 1E-9 M**

3

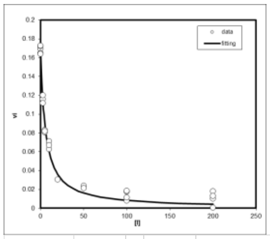

[E] =	1	±	1.92	* 1E-9 M
v_0 =	0.167	±	0.003	* 1E+0 mol.s-1
n =	25			
**K_i =**	**2.469**	**±**	**0.441**	*** 1E-9 M**

4

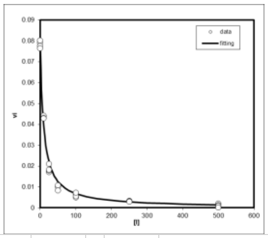

[E] =	1	±	3.202	* 1E-9 M
v_0 =	0.079	±	0.001	* 1E+0 mol.s-1
n =	27			
**K_i =**	**4.394**	**±**	**0.581**	*** 1E-9 M**

5

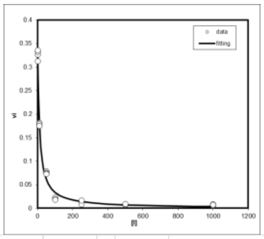

[E] =	1	±	2.831	* 1E-9 M
v_0 =	0.327	±	0.005	* 1E+0 mol.s-1
n =	26			
**K_i =**	**5.295**	**±**	**0.585**	*** 1E-9 M**

6

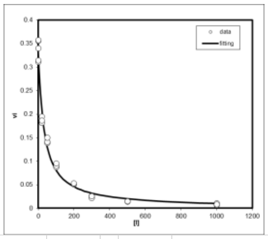

[E] =	1	±	8.881	* 1E-9 M
v_0 =	0.331	±	0.006	* 1E+0 mol.s-1
n =	32			
**K_i =**	**14.97**	**±**	**1.77**	*** 1E-9 M**

7

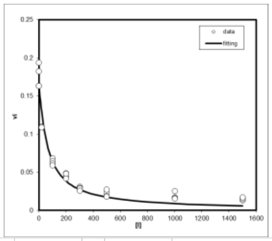

[E] =	1	±	12.85	* 1E-9 M
v_0 =	0.129	±	0.001	* 1E+0 mol.s-1
n =	34			
**K_i =**	**32.1**	**±**	**2.3**	*** 1E-9 M**

8

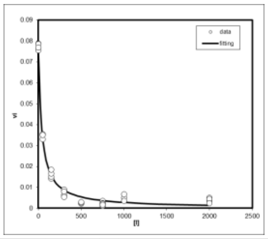

[E] =	1	±	10.42	* 1E-9 M
v_0 =	0.078	±	1E-03	* 1E+0 mol.s-1
n =	32			
**K_i =**	**17.75**	**±**	**1.7**	*** 1E-9 M**

AZA

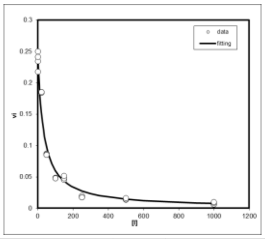

[E] =	4	±	13.51	* 1E-9 M
v_0 =	0.243	±	0.007	* 1E+0 mol.s-1
n =	30			
**K_i =**	**14.94**	**±**	**2.507**	*** 1E-9 M**

U104

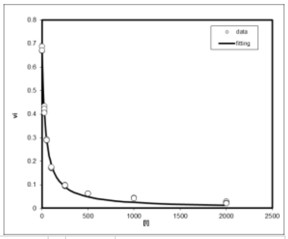

[E] =	1	±	5.478	* 1E-9 M
v_0 =	0.677	±	0.007	* 1E+0 mol.s-1
n =	29			
**K_i =**	**18.1**	**±**	**1.149**	*** 1E-9 M**

Figure S4: Inhibition of CA IX by rising concentration of compounds **1-8**. Plots show the dose dependent decrease of catalytic activity expressed as speed of decrease in absorbance (v_i expressed in $\Delta A.s^{-1}$). The concentration of compound [I] in shown in $nmol.l^{-1}$. Each circle represents one measurement, black line shows fitted curves by the nonlinear least-squares method using Williams-Morrison equation. Enzyme concentration [E], initial velocity v_0, number of measurements (n) and K_i value are shown below each plot. Two know inhibitors of CAs were uses as controls:

AZA	2
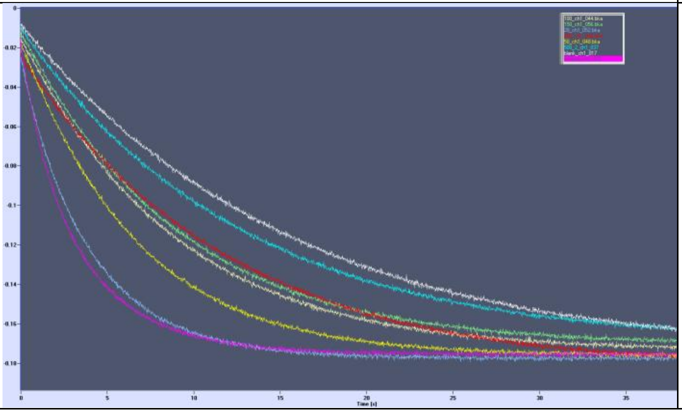	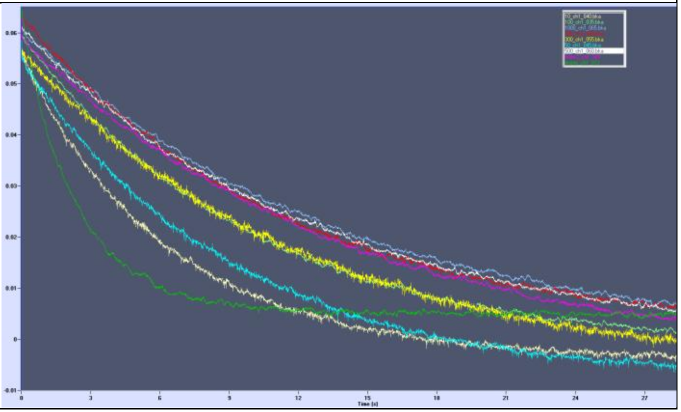

Figure S5: Raw data curves from stopped flow activity assay for CAIX isoenzyme. The absorbance decrease as a function of time in reactions containing increasing concentrations of inhibitors: compound acetacolamide (AZA) and compound **2.**

Inhibition of CA VII

CAVII inhibition

1

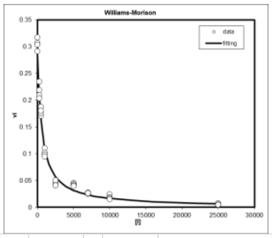

[E] =	13	± 122.6	* 1E-9 M
v_0 =	0.308	± 0.005	* 1E+0 mol.s-1
n =	36		
K_i =	**277**	**± 27.4**	*** 1E-9 M**

2

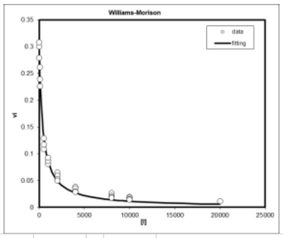

[E] =	14	± 107	* 1E-9 M
v_0 =	0.296	± 0	* 1E+0 mol.s-1
n =	30		
K_i =	**210.4**	**± 22.02**	*** 1E-9 M**

3

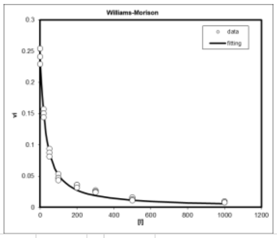

[E] =	14	± 5.575	* 1E-9 M
v_0 =	0.241	± 0.004	* 1E+0 mol.s-1
n =	27		
K_i =	**12.78**	**± 1.133**	*** 1E-9 M**

4

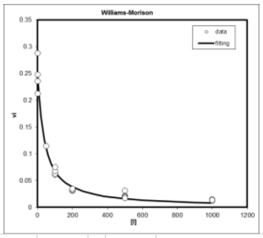

[E] =	14	± 32.42	* 1E-9 M
v_0 =	0.246	± 0.007	* 1E+0 mol.s-1
n =	22		
K_i =	**17.86**	**± 4.814**	*** 1E-9 M**

5

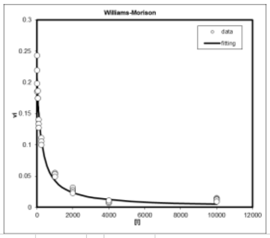

[E] =	13.3	± 96.6	* 1E-9 M
v_0 =	0.207	± 0.006	* 1E+0 mol.s-1
n =	29		
K_i =	**132.7**	**± 24.59**	*** 1E-9 M**

6

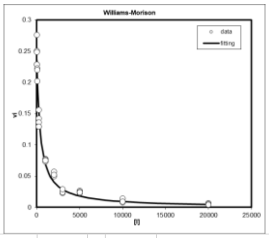

[E] =	13.3	± 105.3	* 1E-9 M
v_0 =	0.243	± 0.005	* 1E+0 mol.s-1
n =	38		
K_i =	**209.2**	**± 23.28**	*** 1E-9 M**

7

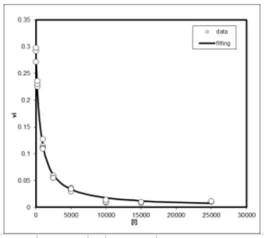

[E] =	1	± 118	* 1E-9 M
v_0 =	0.291	± 0.004	* 1E+0 mol.s-1
n =	27		
K_i =	**343.6**	**± 25.77**	*** 1E-9 M**

8

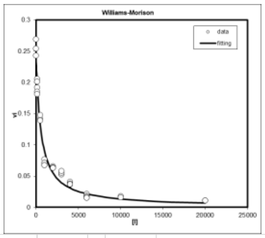

[E] =	13	± 207.4	* 1E-9 M
v_0 =	0.241	± 0.007	* 1E+0 mol.s-1
n =	36		
K_i =	**309**	**± 40.06**	*** 1E-9 M**

AZA

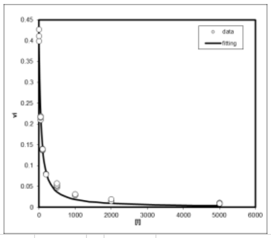

[E] =	14	± 10.81	* 1E-9 M
v_0 =	0.411	± 0.005	* 1E+0 mol.s-1
n =	30		
K_i =	**25.36**	**± 2.273**	*** 1E-9 M**

U104

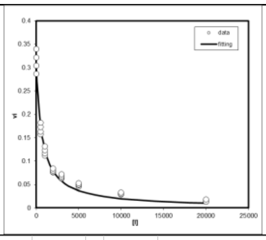

[E] =	14	± 191	* 1E-9 M
v_0 =	0.31	± 0.006	* 1E+0 mol.s-1
n =	32		
K_i =	**354.7**	**± 40.62**	*** 1E-9 M**

Figure S6: Inhibition of CA VII by rising concentration of compounds **1-8**. Plots show the dose dependent decrease of catalytic activity expressed as speed of decrease in absorbance (v_i expressed in $\Delta A.s^{-1}$). The concentration of compound [I] in shown in $nmol.l^{-1}$. Each circle represents one measurement, black line shows fitted curves by the nonlinear least-squares method using Williams-Morrison equation. Enzyme concentration [E], initial velocity v_0, number of measurements (n) and K_i value are shown below each plot. Two know inhibitors of CAs were uses as controls: acetazolamide (AZA) and 4–[[[(4–Fluorophenyl)amino] carbonyl]amino] benzenesulfonamide (U104).

AZA	2

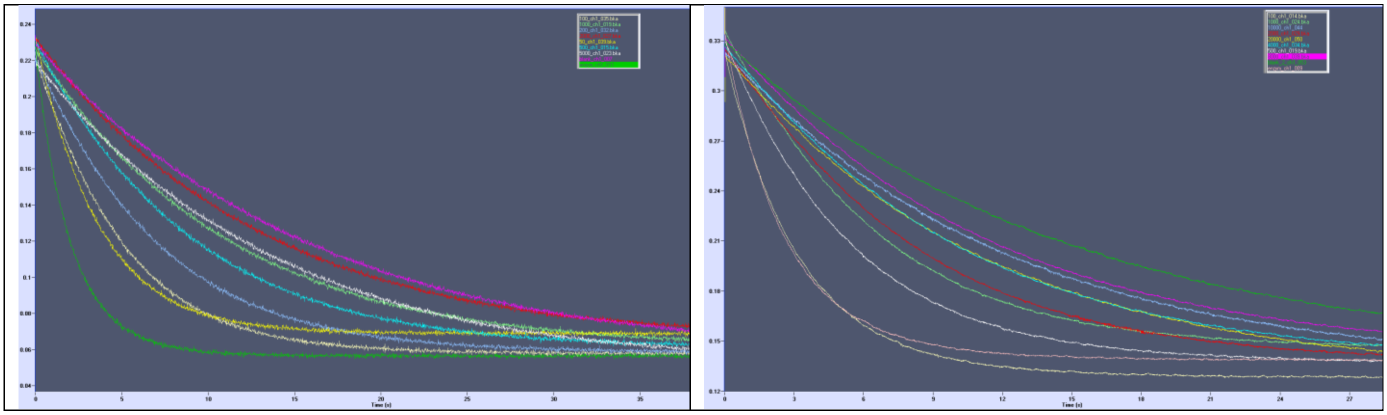

Figure S7: Raw data curves from stopped flow activity assay for CAVII isoenzyme. The absorbance decrease as a function of time in reactions containing increasing concentrations of inhibitors: compound acetacolamide (AZA) and compound **2.**

Structure of inhibitors bound to the active site of CA II

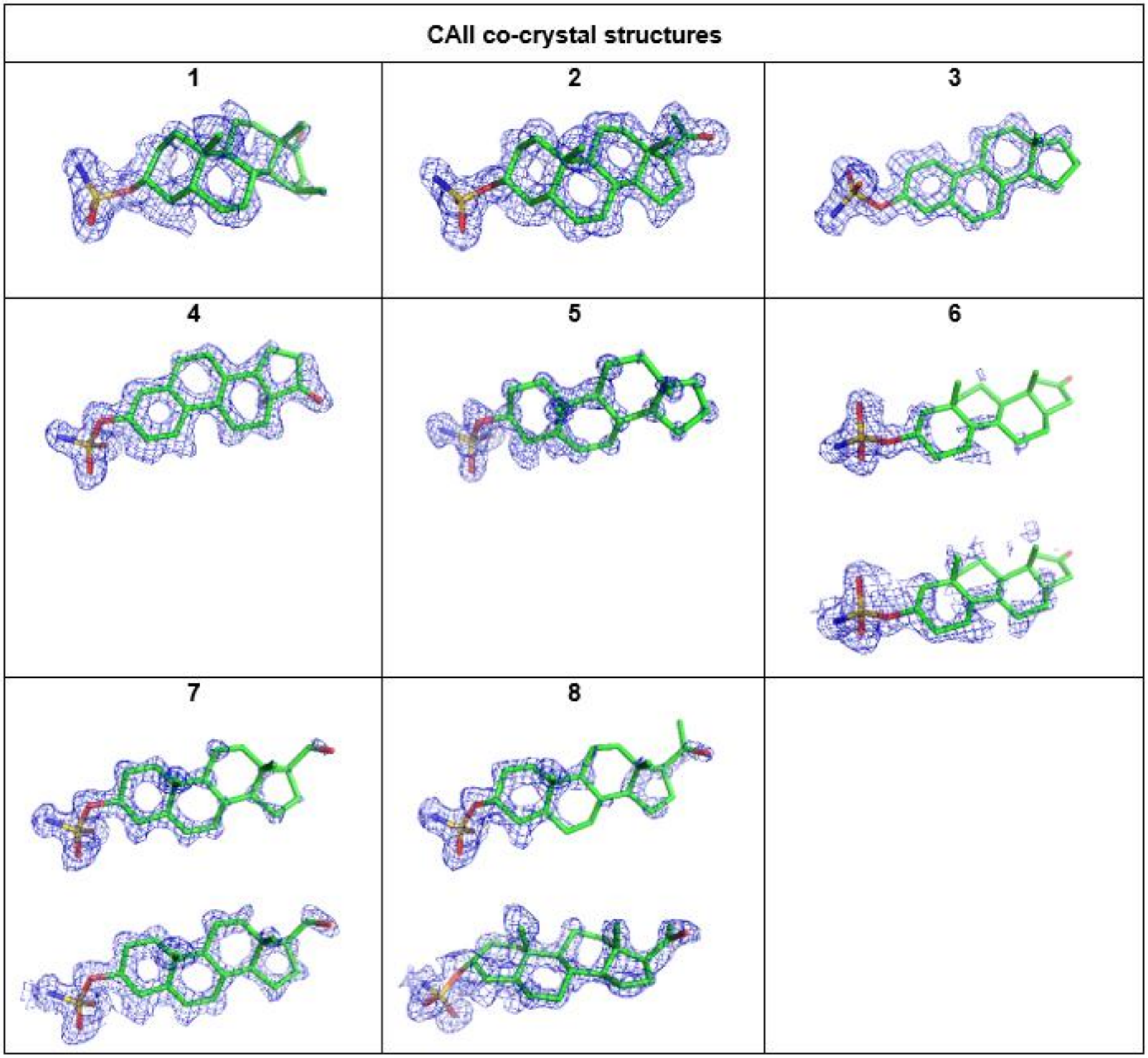

Figure S4. Structure of inhibitors bound to the active site of CA II. Carbon atoms are colored by different colors and oxygen, nitrogen, and sulfur atoms are shown in red, blue, and orange, respectively. $2F_o$-F_c map contoured at 1.5 σ as a mesh. Additionally, lower panel shows $2F_o$-F_c map contoured at 1.0 σ for compounds **6**, **7** and **8**. All compounds were modelled in one conformation with full occupancy except for compound **7** that was modeled with occupancy factor 0.6.

Structure of inhibitors bound to the active site of CA IX-mimic

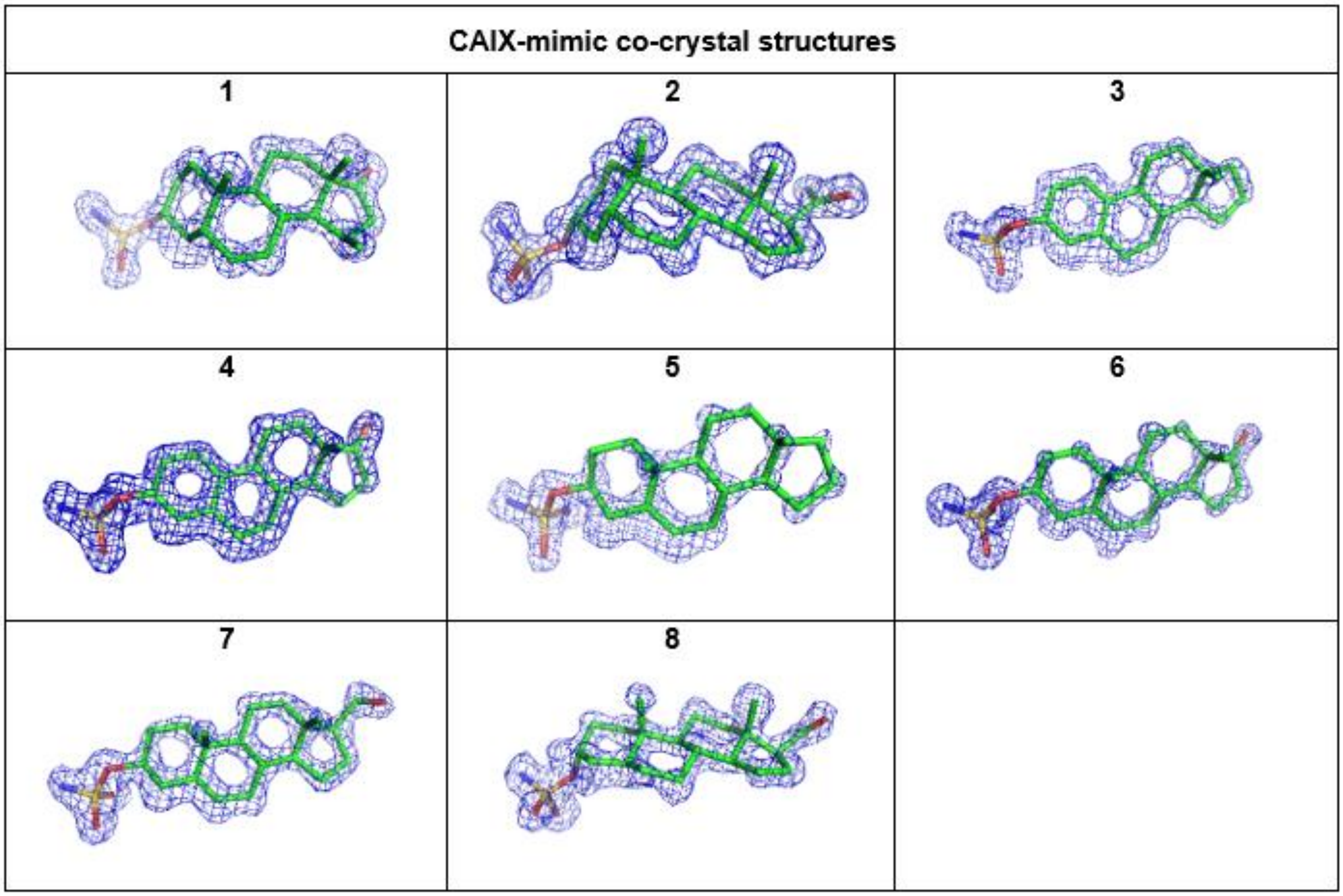

Figure S5. Structure of inhibitors bound to the active site of CA IX-mimic. Carbon atoms are colored by different colors and oxygen, nitrogen, and sulfur atoms are shown in red, blue, and orange, respectively. $2F_o$-F_c map contoured at 1.5 σ as a mesh. All compounds were modelled in one conformation with full occupancy.

Table S1. Diffraction data collection and refinement statistics of the CAII complexes

CAII								
**Ligand**	AKI-33	VK4	BS-1982	VK-42	AKI-2	AKI-1	AKI-13	AKI-12
	**1**	**2**	**3**	**4**	**5**	**6**	**7**	**8**
**Data collection statistics**								
Space group	*P*2₁	*P*2₁	*P*2₁	*P*2₁	*P*2₁	*P*2₁	*P*2₁	*P*2₁
Cell parameters (Å; °)	42.16 41.48 72.07 90.00 104.39 90.00	42.14 41.37 72.08 90.00 104.39 90.00	42.24 41.37 71.80 90.00 104.41 90.00	42.19 41.40 71.89 90.00 104.37 90.00	42.25 41.27 72.37 90.00 104.37 90.00	42.13 41.17 72.33 90.00 104.44 90.00	42.15 41.34 72.14 90.00 104.29 90.00	42.18 41.31 72.31 90.00 104.42 90.00
Wavelength (Å)	1.541870	1.541870	1.541870	1.541870	0.9184	1.541870	1.541870	1.541870
Resolution (Å)	50.0 -1.41 (1.45-1.41)	50.0 -1.4 (1.44 - 1.40)	50.0 - 1.45 (1.49 - 1.45)	50.0 – 1.35 (1.39 – 1.35)	50.0 – 1.0 (1.03 – 1.00)	50.0 – 1.50 (1.54- 1.50)	50.0 – 1.27 (1.30–1.27)	50.0 – 1.4 (1.44–1.40)
Number of unique reflections	39910 (683)	41309 (890)	39046 (1272)	46755 (952)	130194 (9492)	37856 (2517)	60710 (1921)	46643 (3260)
Multiplicity	3.5 (1.1)	3.4 (1.2)	3.8 (1.3)	3.2 (1.1)	3.4(1.7)	3.4 (2.3)	2.9 (1.3)	3.1 (2.2)
Completeness (%)	85.1 (19.9)	86.7 25.3)	91.1 (40.8)	88.1 (24.3)	96.2 (72.3)	97.7 (87.7)	94.9 (40.9)	97.6 (93.5)
R_{merge} [a]	0.030 (0.159)	0.054 (0.309)	0.03 (0.21)	0.021 (0.15)	0.072 (0.851)	0.040 (0.482)	0.030 (0.152)	0.022 (0.130)
$CC_{(1/2)}$ (%) [b]	99.9 (98.1)	99.0 (81.2)	100.0 (90.2	100.0 (96.0)	98.0 (65.0)	99.9 (57.3)	99.9 (94.3)	100.0 (96.6)
Average I/s(I)	25.1 (2.97)	12.5 (1.5)	25.5 (3.3)	30.5 (3.46)	15.2 (0.7)	17.8 (2.4)	18.7 (3.05)	27.2 (5.2)
Wilson B (Å²)	9.8	11.4	11.7	10.4	10.9	13.2	10.4	9.4
**Refinement statistics**								
Resolution range (Å)	39.83 -1.41 (1.45-1.41)	35.59 – 1.40 (1.44 – 1.40)	31.96 – 1.45 (1.49 – 1.45)	32.0 – 1.35 (1.39 – 1.35)	39.92 – 1.00 (1.026 – 1.0)	31.9 – 1.5 (1.54 – 1.50)	35.6 – 1.27 (!.30-1.27)	25.5 - 1.40 (1.44 – 1.40)
No. of reflections in working set	37913 (645)	38249 (690)	37091 (1201)	45466 (916)	126939 (9256)	35968 (1894)	57674 (1819)	44892 (2389)
No. of reflections in test set	1996 (34)	2014 (37)	1953 (64)	1407 (29)	3255 (238)	2358 (124)	3036 (96)	3276 (181)
R value (%) [c]	16.1 (26.2)	16.3 (23.9)	15.7 (22.9)	15.7 (18.4)	16.8 (40.2)	18.2 (31.7)	13.6 (18.8)	17.7 (23.8)
R_{free} value (%) [d]	18.5 (33.7)	19.0 (24.7)	17.9 (26.4)	17.6 (22.5)	17.9 (40.9)	19.7 (33.0)	16.9 (21.7)	19.9 (24.9)
RMSD bond length (Å)	0.009	0.010	0.010	0.011	0.011	0.012	0.090	0.009
RMSD angle (°)	1.6	1.6	1.5	1.6	1.65	1.8	1.5	1.5
Number of atoms in AU (protein/ligand/water molecules)	2135/26/298	2131/27/268	2128/23/245	2158/24/273	2120/24/294 96.0	2122/26/248	2169/28/324	2130/27/300
Mean B value (Å²)	12.5	15.7	15.5	14.0	18.0	18.3	16.5	12.8797
**Ramachandran plot statistics** [e,31,5]								
Residues in favored regions (%)	97	96	96	96	96	96	96	97
Residues in allowed regions (%)	3	4	4	4	4	4	4	3
PDB code	8OMP	8OMN	8OMH	8OMB	8OKQ	8OLM	8OLK	8OLI

The data in parentheses refer to the highest-resolution shell.

[a] R_{merge} = (|I_{hkl} - ⟨I⟩)/I_{hkl}, where the average intensity ⟨I⟩ is taken over all symmetry equivalent measurements and I_{hkl} is the measured intensity for any given reflection

[b] $CC_{(1/2)}$ is the correlation coefficient between random half data sets and from its value the Pearson correlation coefficient of the true level of signal can be calculated: $CC^{*} = \sqrt{2CC_{1/2}/1 + CC_{1/2}}$ [1]

[c] R-value = ||F_o| - |F_c||/|F_o|, where F_o and F_c are the observed and calculated structure factors, respectively

[d] R_{free} is equivalent to R-value but is calculated for 5% of the reflections chosen at random and omitted from the refinement process [2]

[e] As determined by Molprobity [3]

Table S2. Diffraction data collection and refinement statistics of the CAIX-mimic complexes

CAIX-mimic in complex with								
**Ligand**	AKI-33	VK4	BS-1982	VK-42	AKI-2	AKI-1	AKI-13	AKI-12
	**1**	**2**	**3**	**4**	**5**	**6**	**7**	**8**
**Data collection statistics**								
Space group	$P2_1$	$P2_1$	$P2_1$	$P2_1$	$P2_1$	$P2_1$	$P2_1$	$P2_1$
Cell parameters (Å; °)	41.83<br>41.20<br>72.25<br>90.00<br>103.77<br>90.00	41.82<br>41.11<br>72.20<br>90.00<br>103.797<br>90.00	41.84<br>41.18<br>71.91<br>90.00<br>103.88<br>90.00	41.78<br>41.14<br>71.90<br>90.00<br>103.786<br>90.00	41.78<br>41.14<br>72.22<br>90.00<br>103.89<br>90.00	41.870<br>41.210<br>72.200<br>90.00<br>103.85<br>90.00	41.920<br>41.230<br>72.400<br>90.00<br>103.94<br>90.00	41.67<br>41.05<br>71.55<br>90.00<br>103.73<br>90.00
Wavelength (Å)	1.541870	1.541870	1.541870	1.541870	1.541870	0.91840	1.541870	1.541870
Resolution (Å)	50.0–1.25 (1.28-1.25)	50.0 – 1.40 (1.44 – 1.40)	50.0 – 1.45 (1.49 - 1.45)	50.0 – 1.50 (1.54- 1.50)	50.0 – 1.25 (1.28 – 1.25)	50.0 -1.05 (1.08 -1.05)	50.0 – 1.45 (1.49 - 1.45)	50.0 – 1.40 (1.42- 1.40)
Number of unique reflections	62780 (2125)	40088 (1425)	39450 (1524)	35718 (2218)	59060 (2586)	111393 (8132)	39646 (1595)	46311 (2249)
Multiplicity	3.3 (1.4)	3.2 (1.5)	5.2 (1.7)	3.0(1.8)	2.8 (1.6)	6.3 (2.1)	4.9 (1.9)	4.2 (3.0)
Completeness (%)	94.9 (43.6)	85.0 (41.1)	93.0 (48.9)	93.4 (78.9)	89.3 (53.0)	99.5 (97.2)	92.5 (50.5)	99.6 (98.1)
R_{merge} [a]	0.037 (0.383)	0.027 (0.259)	0.04 (0.397)	0.037 (0.681)	0.032(0.790)	0.071 (2.0)	0.065(0.83)	0.06 (0.39)
$CC_{(1/2)}$ (%) [b]	100.0 (70.1)	99.9 (85.4)	100.0 (69.9)	98.5 (83.2)	99.9 (48.4)	99.9 (31.0)	99.9 (48.4)	99.8 (78.6)
Average I/s(I)	17.4 (1.5)	23.9 (2.41)	23.2 (2.0)	21.2 (3.09)	14.7 (0.9)	10.76 (0.7)	14.4 (0.9)	13.5 (2.8)
Wilson B (Å²)	11.0	10.4	12.6	11.6	12.8	12.0	13.44.9	9.3
**Refinement statistics**								
Resolution range (Å)	39.5-1.25 (1.28-1.25)	40.6 -1.4 (1.44 – 1.40)	40.6 -1.45 (1.49 – 1.45)	40.0 – 1.50 (1.54 – 1.50)	40.0 – 1.25 (1.28 – 1.25)	40.65 – 1.05 (1.08 – 1.05)	39.6 – 1.45 (1.49 – 1.45)	26.5 – 1.4 (1.44 – 1.4)
No. of reflections in working set	59641 (2015)	38083 (1346)	37484 (1451)	33609 (2078)	56110 (2444)	109165 (7970)	37663 (1506)	44023 (3199)
No. of reflections in test set	3139 (106)	2005 (71)	1073 (76)	1769 (109)	2954 (129)	2228 (162)	1983 (80)	2271 (150)
R value (%) [c]	13.2 (22.4)	14.6 (21.6)	15.6 (27.5)	15.7 (25.6)	14.3 (30.7)	14.3 (32.8)	16.8 (31,7)	16.3 (22.3)
R_{free} value (%) [d]	16.5 (25.3)	17.0 (21.8)	17.7 (29.0)	17.7 (26.5)	17.4 (31.8)	17.0 (35.4)	18.1 (33.6)	18.4 (25.5)
RMSD bond length (Å)	0.010	0.010	0.011	0.011	0.011	0.011	0.011	0.011
RMSD angle (°)	1.6	1.7	1.6	1.6	1.7	1.7	2.2	1.6
Number of atoms in AU (protein/ligand/water molecules)	2128/26/2 77	2173/27/319	2140/23/276	2170/24/321	2228/24/240	2151/25/318	2131/26/230	2121/27/265
Mean B value (Å²)	15.1	14.0	16.4	16.0	15.4	15.0	19.8	14.0
**Ramachandran plot statistics** [e]								
Residues in favored regions (%)	95.9	97	96	97	95	96	96	97
Residues in allowed regions (%)	5.0	3	4	3	5	4	4	3
PDB code	8OKP	8OLA	8OLF	8OKT	8OKO	8OKE	8OKG	8OKJ

The data in parentheses refer to the highest-resolution shell.

[a] R_{merge} = (|I_{hkl} - ⟨I⟩)/I_{hkl}, where the average intensity ⟨I⟩ is taken over all symmetry equivalent measurements and I_{hkl} is the measured intensity for any given reflection

[b] $CC_{(1/2)}$ is the correlation coefficient between random half data sets and from its value the Pearson correlation coefficient of the true level of signal can be calculated: $CC^* = \sqrt{2CC_{1/2}/1 + CC_{1/2}}$ [1]

[c] R-value = ||F_o| - |F_c||/|F_o|, where F_o and F_c are the observed and calculated structure factors, respectively

[d] R_{free} is equivalent to R-value but is calculated for 5% of the reflections chosen at random and omitted from the refinement process [2]

[e] As determined by Molprobity [3]

Supplementary references

1. Karplus, P. A. & Diederichs, K. (2012) Linking crystallographic model and data quality., *Science.* **336**, 1030-1033.
2. Brünger, A. T. (1992) Free R value: a novel statistical quantity for assessing the accuracy of crystal structures, *Nature.* **355**, 472-475.
3. Chen, V. B., Arendall, W. B., 3rd, Headd, J. J., Keedy, D. A., Immormino, R. M., Kapral, G. J., Murray, L. W., Richardson, J. S. & Richardson, D. C. (2010) MolProbity: all-atom structure validation for macromolecular crystallography, *Acta Crystallogr D Biol Crystallogr.* **66**, 12-21.
